# Acceleration Data Reveal Highly Individually Structured Energetic Landscapes in Free-Ranging Fishers *(Pekania pennanti)*

**DOI:** 10.1371/journal.pone.0145732

**Published:** 2016-02-03

**Authors:** Anne K. Scharf, Scott LaPoint, Martin Wikelski, Kamran Safi

**Affiliations:** 1 Department of Migration and Immuno-ecology, Max Planck Institute for Ornithology, Radolfzell, Germany; 2 Department of Biology, University of Konstanz, Konstanz, Germany; Centre National de la Recherche Scientifique, Centre d'Etudes Biologiques de Chize, FRANCE

## Abstract

Investigating animal energy expenditure across space and time may provide more detailed insight into how animals interact with their environment. This insight should improve our understanding of how changes in the environment affect animal energy budgets and is particularly relevant for animals living near or within human altered environments where habitat change can occur rapidly. We modeled fisher (*Pekania pennanti*) energy expenditure within their home ranges and investigated the potential environmental and spatial drivers of the predicted spatial patterns. As a proxy for energy expenditure we used overall dynamic body acceleration (ODBA) that we quantified from tri-axial accelerometer data during the active phases of 12 individuals. We used a generalized additive model (GAM) to investigate the spatial distribution of ODBA by associating the acceleration data to the animals' GPS-recorded locations. We related the spatial patterns of ODBA to the utilization distributions and habitat suitability estimates across individuals. The ODBA of fishers appears highly structured in space and was related to individual utilization distribution and habitat suitability estimates. However, we were not able to predict ODBA using the environmental data we selected. Our results suggest an unexpected complexity in the space use of animals that was only captured partially by re-location data-based concepts of home range and habitat suitability. We suggest future studies recognize the limits of ODBA that arise from the fact that acceleration is often collected at much finer spatio-temporal scales than the environmental data and that ODBA lacks a behavioral correspondence. Overcoming these limits would improve the interpretation of energy expenditure in relation to the environment.

## Introduction

Habitat change, and ultimately loss, is an ongoing process and the main threat to biodiversity globally [1]. Habitat changes affect animals at an individual level [2,3]. Studies on different mammal species have shown, for example, that individuals living in more fragmented habitats experienced greater physiological stress, and showed differences in behavior and in home range size, when compared to those living in a less fragmented habitat [4–6]. Changes in the environment, may force animals to adjust their movement behavior. This, in turn, can strongly affect their energy expenditure [7].

An improved understanding of animal energy expenditure would allow researchers to describe how individual space use patterns are affected by changes within the environment. In general, animals should strive to minimize energy expenditure. Studies on pallid sturgeons (*Scaphirhynchus albus*) [8], savannah elephants (*Loxodonta africana*) [9] and humans [10] for example show that, when calculating the energetic cost of moving through the landscape in relation to a specific environmental variable (drag for pallid sturgeons, and slope for savannah elephants and humans), the selected pathways corresponded to the route that required the least energy expenditure. Although these studies focused on specific behaviors and considered only one environmental variable, they consistently reveal spatially influenced variation in energetic costs of the behavior in question.

Accelerometers provide biologists with a unique opportunity to collect detailed information on the activities of animals, yielding information on their behavior and indirectly, energy expenditure. From the data collected by the accelerometers, the overall dynamic body acceleration (ODBA) can be calculated as a proxy for individual energy expenditure [11]. ODBA is based on motion, does not contain any behavioral information *per se*, and is strongly correlated with metabolic costs [12,13]. These data, if associated with GPS locations, allow researchers to estimate the spatial distribution of energy expenditure producing a more comprehensive view of the energy landscape from the perspective of the animal [14,15]. Up to now, several studies have taken advantage of the combination of GPS with acceleration data and ODBA to get a better understanding of how animals optimize energetically costly behaviors. For example, ODBA was used to understand how green turtles (*Chelonia mydas)* [16], imperial shag (*Phalacrocorax atriceps)* [17] and imperial cormorants (*Phalacrocorax atriceps)* [14] optimize their energy expenditure during foraging dives. Amelineau et al. [18] investigated how northern gannets (*Morus bassanus*) optimize their foraging events under different wind conditions. Williams et al. [13] used GPS and acceleration data to reveal how pumas (*Puma concolor*) optimize their energy expenditure during hunting events. These studies all aimed to understand how different species optimize their feeding strategy, in terms of energy expenditure, given the environment they were exposed to, by adjusting their behavior.

Understanding where animals spend their energy in space, without focusing on one specific behavior or environmental characteristic, might provide information on how the animal is interacting, in a more generic way, with the environment it encounters. Therefore, we tested the feasibility of reconstruction of the energy landscape of free-ranging animals along a gradient of urbanization. We used ODBA and GPS-recorded location data from 12 fishers (*Pekania pennanti*), a medium-sized forest-dependent carnivore, to investigate the spatial allocation of energy when the animals were active. We expected that (1) animals spend energy non-randomly in space, which at a landscape level is related to (2) their utilization distribution, i.e., their time spent in a given area. As utilization distributions capture the amount of time spent in a given area, which in turn is at least partly related to how quick animals move through space, we hypothesized that animals spend comparably less energy in the core areas of their utilization distributions. The non-random distribution of energy expenditure hypothesized to correlate with utilization distribution is ultimately mediated through non-random use of the environment, hence (3) we also expected a relationship between energy expenditure and environmental characteristics. Given the expected negative relationship between time spent in an area, expressed in the utilization distribution probability, and the energy expenditure mediated through environmental factors, we therefore also (4) hypothesized that the amount of energy expended in an area and that area’s habitat suitability should correlate. Finally, we expected that (5) resources as well as the distribution of utilization distribution core areas should have a patchier distribution with increasing urbanization and affect the movement behavior and energy expenditure of the fishers. Higher proportions of urban area within an animals home range should translate into a more heterogeneous spatial pattern of energy expenditure. We expect this as some activities could be restricted to particular patches that are spatially more restricted than in an area with a more homogeneous landscape. By combining the acceleration data with spatial information, we aim to directly translate habitat properties as assessed by remote sensing, such as resource composition or availability, into energetic costs for naturally behaving animals and thus obtain better insight into the animals' interaction with its environment.

## Material and Methods

### Study area and tracking data

**Fig 1:**
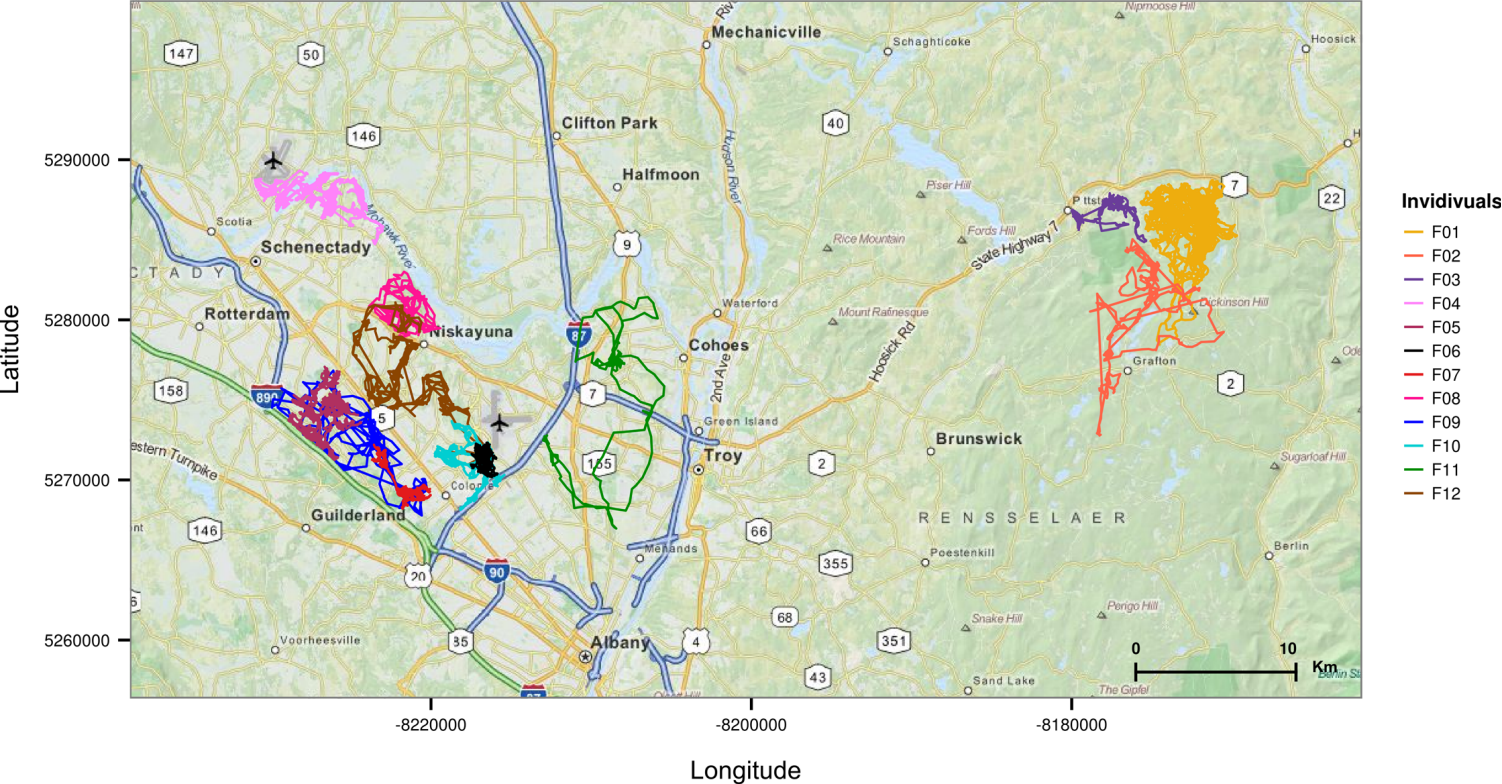
Tracks of the individuals included in this study.

Twelve fishers were tracked near Albany (New York, USA) during 2009–2011 (Fig 1, Table 1). Nine of these individuals were tracked in suburban forest patches. This 350 km^2^ area is composed of residential and commercial land interspersed with forest patches. It is relatively flat (< 100 m change in elevation) with a road density of 4.77 km/km^2^ [19] and a human population density of 438 persons/km^2^ [20]. The remaining three individuals were tracked in a nearby area (Grafton Lakes State Park, 9.5 km^2^), a mostly contiguous forest containing recreation trails and a few gravel roads (see [21] for details). Capture and handling protocols are described in LaPoint et al. [21].

**Table 1:** General information of the tracked individuals.

				Number of GPS
		% of home range	Deployment duration in	fixes
Individuals	Sex	with urban area	days (time period tracked)	used in analysis
F01	m	0.3	71 (17.03.– 29.05.2011)	3714
F02	m	3.5	24 (04.– 29.12.2009)	708
F03	f	4.0	28 (13.08.– 14.09.2009)	443
F04	m	13.3	49 (09.02.– 02.04.2010)	2669
F05	f	15.6	18 (16.12.2010–05.01.2011)	913
F06	f	16.5	16 (21.01.– 08.02.2011)	423
F07	m	27.9	18 (23.12.2010–12.01.2011)	684
F08	f	35.7	19 (11.02.– 04.03.2011)	765
F09	m	36.3	20 (11.02.– 05.03.2009)	655
F10	m	43.0	22 (19.01–12.02.2011)	737
F11	m	49.9	10 (08.– 18.03.2011)	617
F12	m	50.9	24 (10.02.– 08.03.2011)	1253

Fishers were fitted with tracking collars equipped with GPS and tri-axial accelerometers (E-obs GmbH; Grünwald, Germany). The collars recorded a GPS-location every 10 minutes for five individuals and every 15 minutes for one individual. The GPS collars of the remaining six individuals, were programmed with a dynamic sampling, taking GPS fixes every two minutes when the animal was highly active (e.g., running), every 10 minutes at moderate activity, and every 60 minutes during low activity (e.g., resting) (S1 Table) [22]. We regularized the location data for the individuals with dynamic sampling to compensate for different sampling schedules and to obtain an unbiased quantification of activity levels. To do this we created locations with the same coordinates every 10 minutes during inactive periods. Similarly, we subsampled the locations by a minimum of 10 minutes when the fisher had been active and the collar collected locations every two minutes. The accelerometer data were recorded at 18.74Hz in a 3.5-second burst every 3 minutes, obtaining for every burst 54 accelerometer measurements. We associated each acceleration burst to the location closest in time (median time gap between the acceleration burst and the GPS fix was 1.2 seconds and the maximum time gap was 60 seconds).

### ODBA calculation

To transform the raw accelerometer data into m/s^2^, we applied the equation provided by the manufacturer of the collars:

$$a_{i}=\left(n_{i}-n_{i,zerog}\right)\cdot c_{i}\cdot g$$

where a_i_ is the acceleration of axis *i* in m/s^2^; *i* is the axis x, y or z; n_i_ is one digital sample of raw data for axis *i*; n_i,zerog_ is the raw value for zero acceleration for axis *i*; c_i_ is the slope for axis *i* and *g* is the magnitude of observed gravitational acceleration caused by the earth (9.81 m/s^2^). The default value for the slope for accelerometers configured with high sensitivity was 0.001, and for those configured with low sensitivity was 0.00269 as indicated by the manufacturer. The default value for zero acceleration was 2048.

We quantified the mean ODBA per burst as in Wilson et al. [11], using the following equation:

$${ODBA}_{j}=\frac{\sum_{i=1}^{n}(|x_{i}-\bar{x}|+|y_{i}-\bar{y}|+|z_{i}-\bar{z}|)}{n}$$

Here, ODBA is calculated for burst *j*. A burst consists of *n* samples in each of the three axes (x, y and z). x_i_ represents i^th^ component and $\bar{x}$ the mean of all *n* samples of the x-axis of burst *j* (same for axis y and z). Due to the different sensitivity settings of the accelerometers (S1 Table), we had to standardize the ODBA values for cross comparability between individuals. We standardized the ODBA to range between 0 and 1 within each individual as follows:

$$ODBA\prime_{i}=\frac{{ODBA}_{i}-min(ODBA)}{max(ODBA)-min(ODBA)}$$

### Landscape data

Land cover data was obtained from the National Land Cover Database 2011 [23] at 30 m resolution. We visually compared the land cover map to the Google Earth satellite images closest in time to the tracking periods of each individual, to account for potential land cover changes that may have occurred since the creation of the land cover data set. We reclassified the original land cover types (see [23,24]) into developed low, developed high, and the natural land use categories deciduous forest, evergreen forest, mixed forest, shrub, grassland, crop, woody wetland, herbaceous wetland, barren, and open water. For each 30 m grid cell of the land cover map, we also calculated the distance to the forest edge and estimated the proportion of urban area and landscape heterogeneity within a 240 m radius circle (S2 Table). We chose this radius as possible distance of perception based on our experience in the field while approaching these individuals for data downloads, where we would observe them move away from us when the distances were less than 240 m. We also included the distance to roads from each grid cell [25]. We quantified the proportion of urban area within each individual's home range (i.e., the 95% of the utilization distribution; see details in *Statistical analyses* below). As urban areas we included those areas that were classified as developed in the land cover map in addition to roads (S2 Table).

### Statistical analysis

As the tracked fishers were highly nocturnal, we identified resting bouts as time periods with low activity levels lasting for more than 4 hours during the day, indicated by low variability in the accelerometer measures [21] and excluded them from our analyses. We also excluded resting periods that met these criteria but extended into the night. Additionally, we excluded the first 48 hours of data collection after collaring to avoid possible effects of capture and handling. Thus, for all subsequent analyses (energy landscape models, utilization distribution, and habitat suitability models) we used only the active data set and, where applicable, regularized location data.

To model ODBA as a function of space and time for each individual, we used generalized additive models (GAM) [26], since we were expecting potentially complex and non-linear spatio-temporal patterns. We fitted the spatial position as an explanatory thin plate regression spline smooth term consisting of the latitude and longitude (of where each burst was collected) to the cubic root of each single ODBA burst. The cubic root transformed the residuals of the model to meet the Gaussian distribution assumption. We set the number of knots, the k value of the smooth term, to 100. For all GAMs we allowed the model to add an extra penalty to each term added and thus, as part of the model fitting, allow to remove terms completely from the model. The distribution family was chosen to be Gaussian and the smooth terms were estimated based on the restricted maximum likelihood, “REML”. To incorporate the temporal pattern in energy expenditure we included the time of the day at which each burst was collected in seconds as a cyclic penalized cubic regression spline smooth term. For this smooth term we set k to 10. The residuals were checked for Gaussian normal distribution and for the absence of auto-correlation to meet the assumptions of the GAM. The models were calculated with the R package *mgcv* [26].

We estimated the proportion of time spent within the different areas of an individual’s home range, the individual utilization distribution (UD), using the dynamic Brownian bridge movement model [27] with the R package *move* [28]. To test whether the predicted energy landscape was correlated with the utilization distribution, we modeled ODBA as a function of spatial position, time and UD for all individuals using a generalized additive mixed model (GAMM) [26]. We extracted the UD value for each location where each ODBA burst was collected and included it as an explanatory variable together with longitude and latitude and time of day as smooth terms as in the previous GAMs. We included individuals as a random factor.

To evaluate the influence of the environment on the spatial distribution of energy expenditure, we added land cover as a categorical variable, distance to forest edge, landscape heterogeneity, proportion of urban area and distance to roads, all as continuous values, to the previous purely spatio-temporal explicit models, and searched for a minimum adequate model for each individual separately. We used Akaike's Information Criterion corrected for small sample sizes (AICc) to rank the models of each individual, selecting models with a delta AICc value lower than 4 (S3 Table), as these are the models that have considerably greater empirical support [29]. We used weighted model averaging on this subset of best models and calculated a prediction of the energy landscape for each individual. The AICc, the weighted model averaging and the prediction were calculated with the R package *MuMIn* [30].

We calculated habitat suitability using a step selection function [31,32]. This function compares the environmental attributes of an observed step (based on two consecutive GPS locations) with a number of random steps that have the same starting point. We generated the random steps from a multivariate normal distribution, using the function *rmvnorm* of the R package *mvtnorm* [33], maintaining the variance/covariance structure of speed and turning angle of the empirical track of each individual. We used 5 random steps per observed step, converting speed to step length by multiplying the random speed by the time between fixes of the corresponding observed step. To analyze the habitat preferences, we compared the environmental characteristics of the end points of each observed step with its corresponding random steps, by means of a conditional logistic regression model using the *mclogit* function of the R package *mclogit* [34]. The environmental variables included in the model were land cover, distance to forest edge, landscape heterogeneity, proportion of urban area and distance to roads (S2 Table). As the likelihood of realizing a specific option is a function of step length and relative turning angle, we also included these two measurements as variables in the model. We built one model per individual, based on 75% of the observed locations, and calculated the predicted habitat suitability. For the predictions, we kept distance and relative turning angle constant, selecting a random pair of values from the previously mentioned multivariate normal distribution. We used the previously excluded 25% of the observed locations to assess the performance of the model predictions by comparing them with random points selected form the obtained maps (for details see S1 Appendix). To test whether the predicted energy landscape was correlated with the habitat suitability, we modeled ODBA as a function of spatial position, time and habitat suitability for all individuals using a GAMM. We extracted the habitat suitability value for each location where each ODBA burst was collected and included it as an explanatory variable together with longitude and latitude and time of day as smooth terms as in the previous GAMs. We included individual as a random factor.

As a measure of heterogeneity in the predicted energy landscape, we used the obtained adjusted R^2^ of the spatio-temporal models. As spatio-temporal non-randomness increases, the spatial and temporal explanatory variables in the GAMs can capture more of the pattern. Therefore the adjusted R^2^ of the models would increase with increasing non-randomness in the distribution of ODBA, i.e. increasing heterogeneity in the energy landscape. To investigate whether urbanization and energy expenditure were correlated we calculated the Pearson's correlation coefficient between the adjusted R^2^ of the spatio-temporal GAMs and the degree of urbanization each individual experienced. We used the adjusted R^2^ as an unbiased estimator which only increases when the addition of explanatory variables improves R^2^ more than expected by chance taking into account the number of additional variables and in case of the smooth terms the number of knots used in the additive models [35]. We also directly tested whether urbanization resulted in more heterogeneity in predicted energy expenditure in the landscape by calculating the spatial variance of the predicted values from the spatio-temporal GAMs, and correlated the predicted values with the degree of urbanization.

In addition, we identified the areas with the most extreme predicted ODBA values. We did this by identifying the hot spots of the lowest (ODBA valleys) and highest (ODBA peaks) energy expenditure for each individual. We defined them as the areas with the lowest 5% and highest 5% of predicted ODBA values respectively. We compared the environmental composition and the time spent in the ODBA valleys versus peaks. For each hot spot type we calculated its area (m^2^), extracted the time spent in it from the UD, and its environmental composition. To compare the time spent between hot spots, we built a linear model where the spent time was the response variable and the type of hot spot, area and individuals were the explanatory variables. As the amount of time spent in a hot spot will depend on its size, we included area in the model and individuals to account for potential differences between them. To compare the environmental composition of the two types of hot spots, we applied a compositional analysis using the function *adonis* from the R package *vegan* [36]. The environmental variables including land cover, distance to forest edge, landscape heterogeneity, proportion of urban area and distance to roads (S2 Table) were the response variable, and type of hot spot and area were the explanatory variables. We included individuals as strata and set the number of permutations to 999.

To investigate if other movement-related behaviors changed along the urbanization gradient, we calculated the number of active bouts per day, the duration of these active bouts and the cumulative distance traveled per day for each individual. We defined “day” as the period of time from sunset to sunset (of the following day). We calculated the bouts of activity from the acceleration data, being each bout a continuous period where the animal was active. Then, we correlated each of these three measurements with the degree of urbanization. We conducted all analysis with R 3.1.0 [37].

## Results

**Fig 2:**
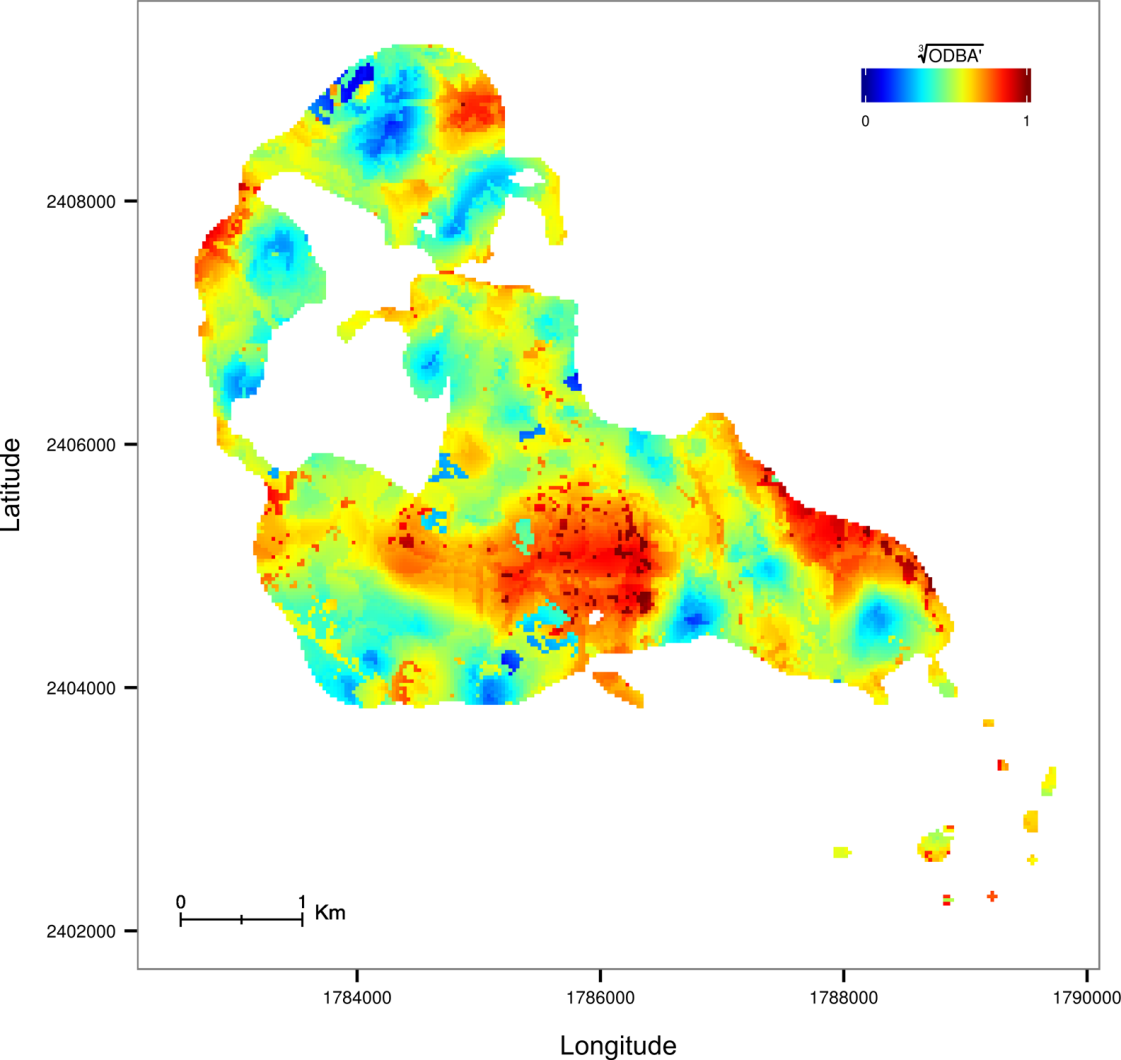
Predicted energy landscape for individual F12. The prediction is made from the averaged set of best models including spatial position, time of day and environmental variables. The area of the map corresponds to the home range of this individual (95%UD).

Our analysis revealed a non-random spatial structure of energy expenditure (Fig 2, S1 Fig). The mean ± SD value of adjusted R^2^ for the models only including the longitude and latitude was 0.35 ± 0.09 (Table 2). For most individuals the time of day did not have a large effect on the distribution of energy, as the adjusted R^2^ of the spatio-temporal models increased only marginally (mean ± SD = 0.37 ± 0.08, Table 2).

**Table 2:** Generalized additive models (GAM) results, of the model including only spatial position, the model including the spatial position and time of day, and the model including also the environmental variables.

Individuals	Adj. R^2^ of spatial model	Adj. R^2^ of spatio—temporal model	Adj. R^2^ of spatio—temporal and environment model	Variance of the predicted values of the spatio—temporal model
F01	0.23	0.26	0.26	0.05
F02	0.47	0.47	0.47	0.77
F03	0.41	0.43	0.43	0.01
F04	0.26	0.31	0.32	0.05
F05	0.38	0.40	0.42	0.06
F06	0.26	0.30	0.30	0.01
F07	0.33	0.35	0.36	0.12
F08	0.46	0.46	0.46	0.07
F09	0.42	0.44	0.44	0.08
F10	0.23	0.25	0.25	0.04
F11	0.34	0.36	0.37	0.03
F12	0.38	0.38	0.40	0.10

**Fig 3:**
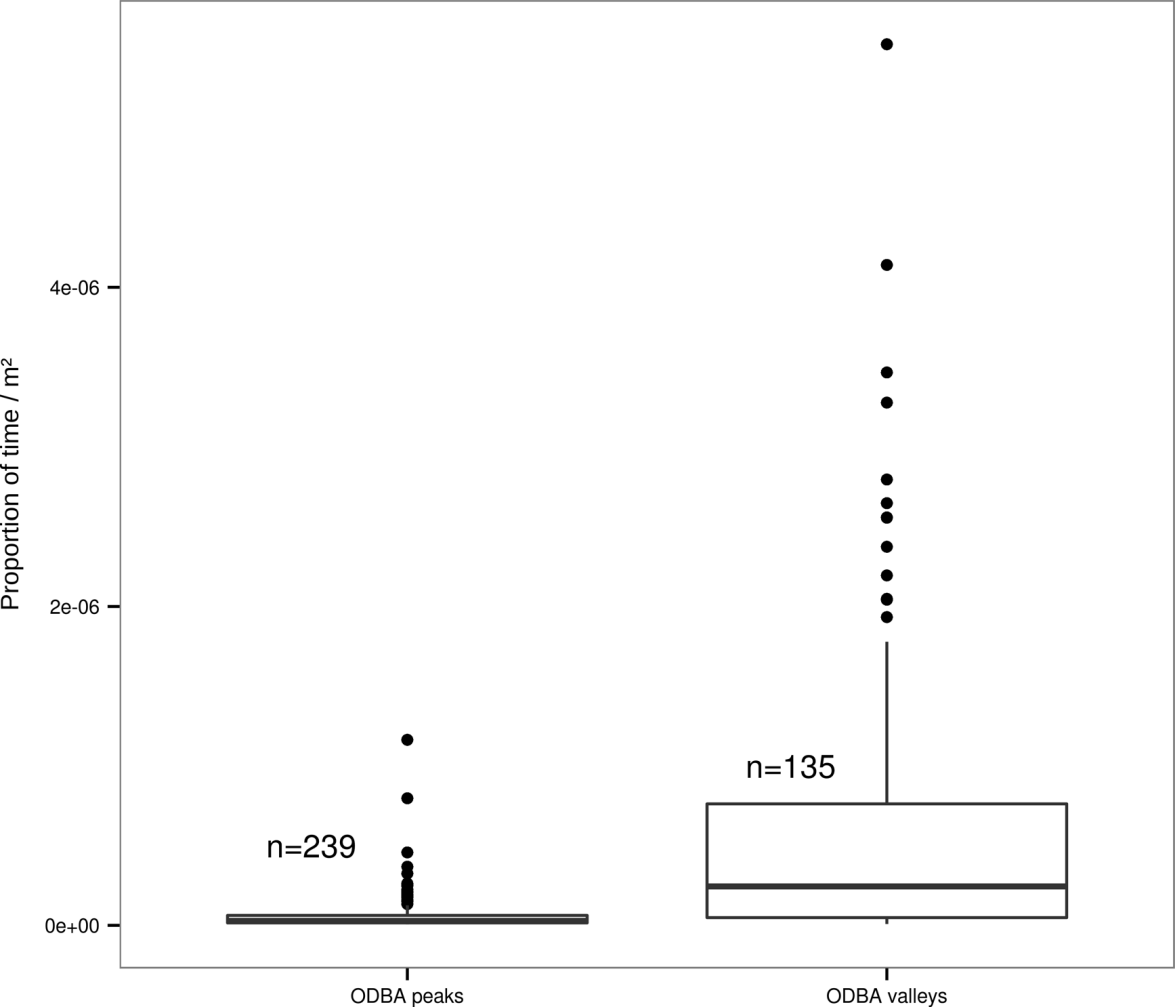
Comparison of time/m^2^ spent in ODBA valleys and ODBA peaks across all individuals. The y-axis represents the proportion of time spent in each hot spot divided by its area. *n* is the total number of each type of hot spot.

The time spent in an area (UD, S2 Fig) had a significant negative influence (estimate ± SE = -20.05 ± 0.5, t-value = -40.03, p<0.001, DF = 13627, adjusted R^2^ = 0.10) on the energy expenditure. This result was supported by the analyses of the time spent in ODBA valleys versus peaks, where we found that fishers spent 0.015 ± 0.002 (estimate ± SE, t-value = 7.26, p<0.001, F_13,360_ = 9.0, Fig 3) times more time in ODBA valleys than in peaks.

The mean ± SD adjusted R^2^ across models after inclusion of environmental variables and subsequent model selection was 0.37 ± 0.08 (Table 2), showing only small increases compared to the first model. Distance to forest edge, percentage of urban area, and distance to roads were retained in the models of all 12 individuals. The remaining environmental variables were retained in variable combinations for each individual (Table 3). The importance, size effect, and sign of the environmental variables varied across individuals (Table 3), yet did not show a consistent pattern related to the urbanization gradient.

**Table 3:** Contribution of the environmental variables included in the GAMs.

Environmental variables	Number of models in which present	Size effect range
Distance to the forest edge	12	-0.1825–0.0263
Proportion of urban area	12	-0.0807–0.0220
Distance to roads	12	-0.4607–0.0935
Landscape heterogeneity	11	-0.1694–0.1871
Land cover *	5	
Developed low		-0.2838–0.3695
Deciduous forest		-0.4166–0.8625
Coniferous forest		-0.5265–0.1197
Mixed forest		-0.3887–0.4793
Shrub		-0.0162–0.1270
Crop		-0.3362–0.2367
Woody wetland		-0.3952–1.0853
Herbaceous wetland		-0.0126 (only present in one model)
Grassland		0.0027 (only present in one model)

* Land cover is included as a factor in the model, all land cover types are compared to the land cover type “Developed high”

The habitat suitability models performed well (S3 Fig). The mean ± SD habitat suitability of the observed locations across individuals was 0.51 ± 0.27 and significantly higher than the average of 0.28 ± 0.26 from the randomly selected points (S1 Appendix for details). We found a negative influence of the habitat suitability (estimate ± SE = -0.12 ± 0.01, t-value = -16.2, p<0.001, DF = 13627, adjusted R^2^ = 0.05) on the energy expenditure. These results were supported by the differences we found between the ODBA valley and peaks in environmental composition (F_1,371_ = 11.97, p<0.001).

The individual fishers had variable proportions of urban area within their home ranges, resulting in a gradient that spanned from 0.3 to 51% (Table 1). Contrary to our expectations, however, the heterogeneity in the energy landscape was not related to the urbanization gradient. The correlation between the adjusted R² of the models and the percentage of urban area in the home range was very low (r = -0.006, DF = 10, p = 0.98). In addition, the differences in the variance of the predicted values of the energy landscape between individuals (Table 2) were not correlated with the percentage of urban area in the home range (r = -0.28, DF = 10, p = 0.38).

**Fig 4:**
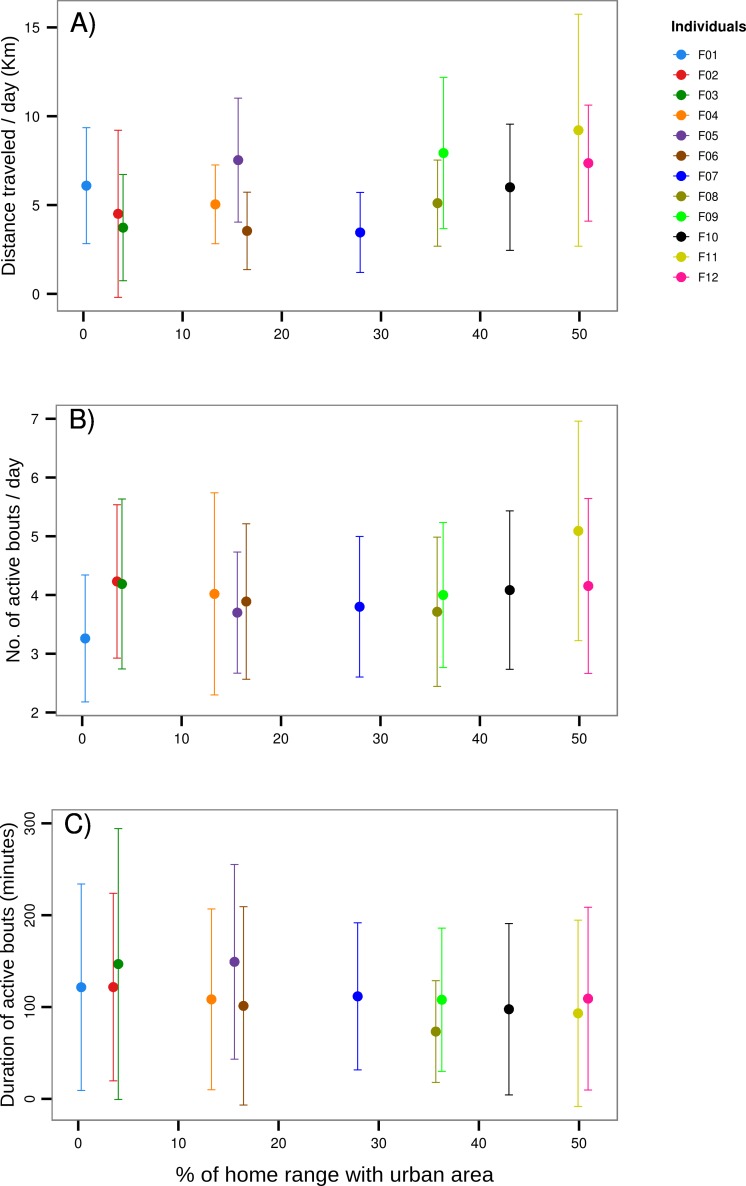
Activity measurements per individual along the urbanization gradient. (A) Cumulative distance traveled per day (mean±SD). (B) Number of active bouts per day (mean±SD). (C) Duration of active bouts (mean±SD).

Individual variation in total daily distance traveled, and the duration and number of activity bouts per day was high. We found a low, but positive relationship between the total distance traveled (slope ± SE = 0.033 ± 0.012, t-value = 2.828, DF = 291, p<0.01) and the degree of urbanization (Fig 4). We also found a low, but significant, relationship between the degree of urbanization and the number of active bouts per day (slope ± SE = 0.012 ± 0.004, t-value = 2.791, DF = 343, p<0.01) and the duration of these active bouts (slope ± SE = -0.651 ± 0.162, t-value = -4.009, DF = 1341, p<0.001, Fig 4).

## Discussion

The fishers we studied spent energy in a spatially structured manner during their active period, that did not depend on the time of day. It seems that fishers tended to spend the same amount of energy in a given area independent of the time of the day, which points to some environmental structuring of energy use. However, our environmental variables, including natural and human related variables, could not explain energy expenditure all too well. As we expected, the amount of time spent in a given area was negatively related with how much energy they spent there. Following this expectation we found that the time spent in the ODBA valleys was significantly longer than in the ODBA peaks. This is intuitive, as an animal moving fast and therefore spending high amounts of energy will implicitly spend less time in that same area. Although the relationship between utilization distribution and energy expenditure was significant, the model only explained a small proportion of the variance in the data.

According to our expectation, composition of the environment should influence activity and therefore shape individual energetic landscapes [38]. However, we were unable to identify the environmental characteristics that defined individual energy landscapes. Nevertheless, we did find a relation between habitat suitability and energy expenditure across all individuals. They spent less energy in areas with higher habitat suitability. We also found that the environmental composition of the ODBA valleys and peaks was significantly different. Although the habitat suitability could only explain a very small part of the variance in energy expenditure across individuals, these results indicate that the environment did influence how much energy these fishers were spending in a given place. Fishers are forest specialists [39] and our simple classification based on remote sensing seems not to incorporate the heterogeneity of forest environments. Smaller scale variation in forest composition and micro-climate likely have important impacts on the behavior of fishers [40], but these could not be measured in this study. Accelerometer-derived data might provide more insight into how animals respond to these subtle nuances, yet there is a large mismatch in the scales at which acceleration data and environmental data are typically collected. If the changes in behavior occur at very fine temporal and spatial scales, the available remotely-sensed environmental data may be insufficient to allow us to detect them.

Contrary to our prediction, neither the total amount of explained variance in the observed energy expenditure, nor the total amount of predicted variance in the energy landscape were explained by the amount of urbanization. We found high inter-individual variation in activity measurements such as duration and number of daily bouts of activity, as well as the cumulative distance traveled per day. Although these measurements were only weakly correlated with the degree of urbanization, individuals with more urban area in their home ranges seem to have slightly shorter activity bouts that are then compensated by a higher number of bouts per day. There also seems to be a slight increase in total distance traveled per day as the percentage of urban area within the home range increased. Despite these results hinting towards some effect of the urbanization on the activity budget, this was not reflected in differences of spatial distribution of energy expenditure. Overall, our results indicate that, if present, the effect of urbanization on the energy landscape are subtle. Either fishers were not as negatively affected by urbanization as one might expect or the effects were not reflected appropriately by ODBA.

One limitation of using ODBA is that it lacks a behavioral context. Although specific behaviors have been identified from tri-axial accelerometers [41–44], doing so remains challenging, in particular teasing apart distinct behaviors that may have similar acceleration characteristics (e.g., hunting or escaping). For a more complete view of the energetic landscape we would need a full cost-benefit comparison, requiring more information, ideally from identified behaviors with known energetic costs.

The survival of animals largely depends on the balance between energy acquisition and expenditure [45]. Understanding where, when, and how much energy animals spend, is key to understanding the interactions of species and individuals with their environment. Our work revealed a spatial structure of energy expenditure and suggests that close examination of environmental details are necessary to understand how the landscape structures energy expenditure. Future efforts should strive to identify the additional factors that underlie the non-random structure that we observed. This may require new data at spatial and temporal resolutions that more clearly match the perspectives of the study animals. Disentangling the causal relationships from which the patterns we observed emerged will improve our understanding of how environmental changes affect animal energy expenditure and behavior, potentially aiding efforts to mitigate the causes and consequences of habitat alteration.

## Supporting Information

S1 Appendix Habitat suitability model performance assessment. (PDF)

S1 Fig Predicted energy landscape for all individuals. The prediction is made from the averaged set of best models, per individual, including spatial position, time of day and environmental variables. The areas of the maps correspond to the home ranges of the individuals (95%UD).
(TIF)

S2 Fig Utilization distribution (UD) of each individual. The color scale represents the relative proportion of time spent in each cell. The areas of the maps correspond to the home ranges of the individuals (95%UD).
(TIF)

S3 Fig Habitat suitability maps of all individuals. The areas of the maps correspond to the home ranges of the individuals (95%UD).
(TIF)

S1 Table Supplementary information of the tracked individuals. (PDF)

S2 Table Environmental variables included in the analyses. (PDF)

S3 Table AICc values of GAMs including environmental variables. For each individual all possible combinations of the environmental variables were tested. Within each individual, the models are sorted by increasing ΔAIC values.
(PDF)
