# Supplementary material for: Acceleration Data Reveal Highly Individually Structured Energetic Landscapes in Free-Ranging Fishers (Pekania pennanti)
Source: PLoS One. 2016 Feb 3;11(2):e0145732. doi: 10.1371/journal.pone.0145732 (PMC4739643; doi:10.1371/journal.pone.0145732)
Supplement: S1 Appendix — (PDF) [file pone.0145732.s001.pdf]

S1 Appendix. Habitat suitability model performance assessment

For each individual we did not include in the model 25% randomly selected observed locations. For these observed locations we extracted the corresponding habitat suitability value from the obtained model. We also extracted, a 100 times, randomly the same number of points as locations excluded from each predicted map within the individuals home range (95%UD). With a Kolmogorov-Smirnov test we compared the distribution of habitat suitability values from the observed locations with those from the random points extracted. For all individuals the two distributions were significantly different (Table 1).

Table 1. Results form the Kolmogorov-Smirnov tests

Individuals	D	p-value
F1	0.11	<0.001
F2	0.26	<0.001
F3	0.22	<0.001
F4	0.28	<0.001
F5	0.17	<0.001
F6	0.23	<0.001
F7	0.16	<0.001
F8	0.37	<0.001
F9	0.32	<0.001
F10	0.32	<0.001
F11	0.58	<0.001
F12	0.40	<0.001
