# Supplementary figures and images for: Acceleration Data Reveal Highly Individually Structured Energetic Landscapes in Free-Ranging Fishers (Pekania pennanti)
Source: PLoS One. 2016 Feb 3;11(2):e0145732. doi: 10.1371/journal.pone.0145732 (PMC4739643; doi:10.1371/journal.pone.0145732)

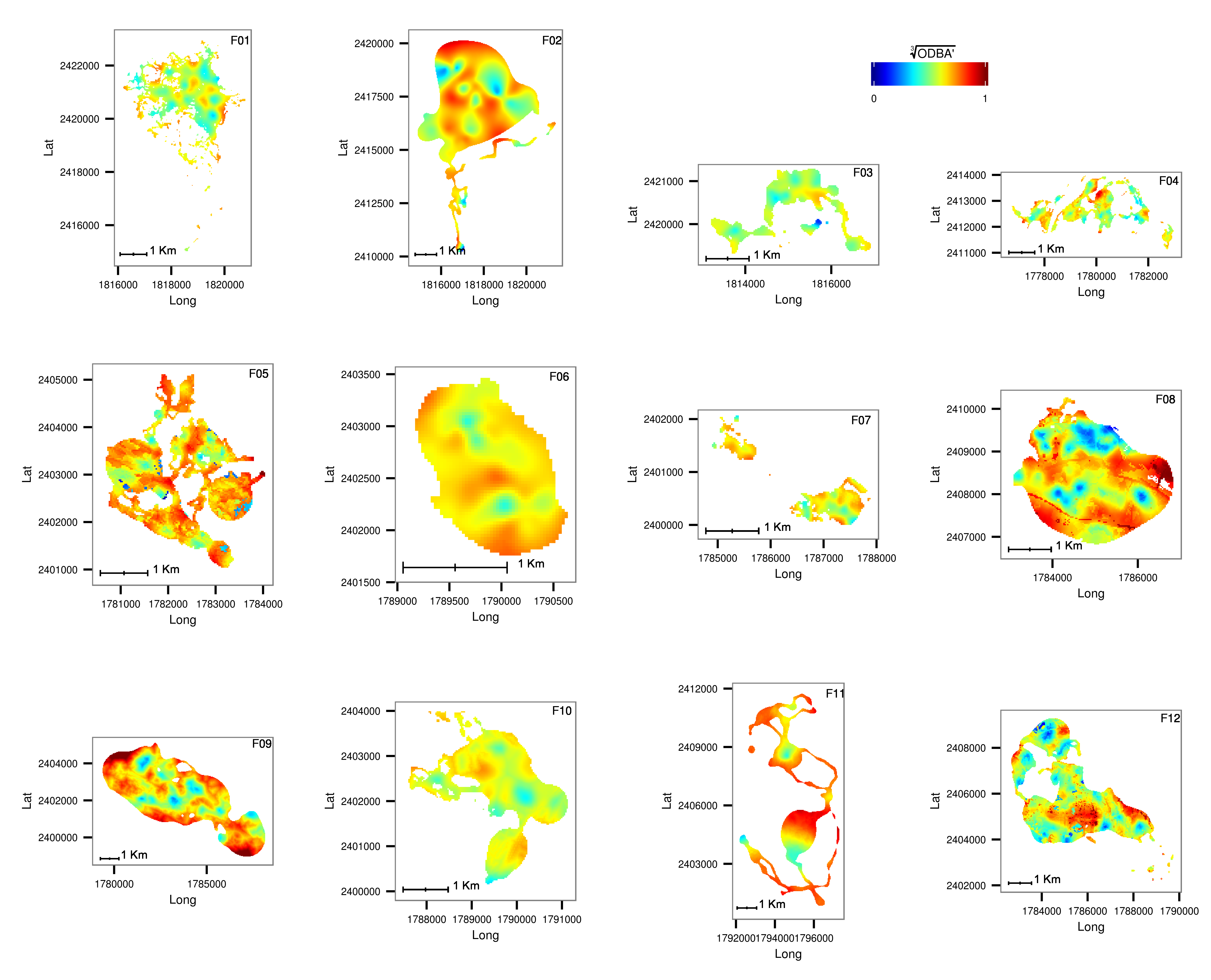

Supplement: S1 Fig — The prediction is made from the averaged set of best models, per individual, including spatial position, time of day and environmental variables. The areas of the maps correspond to the home ranges of the individuals (95%UD). (TIF) [file pone.0145732.s002.tif]

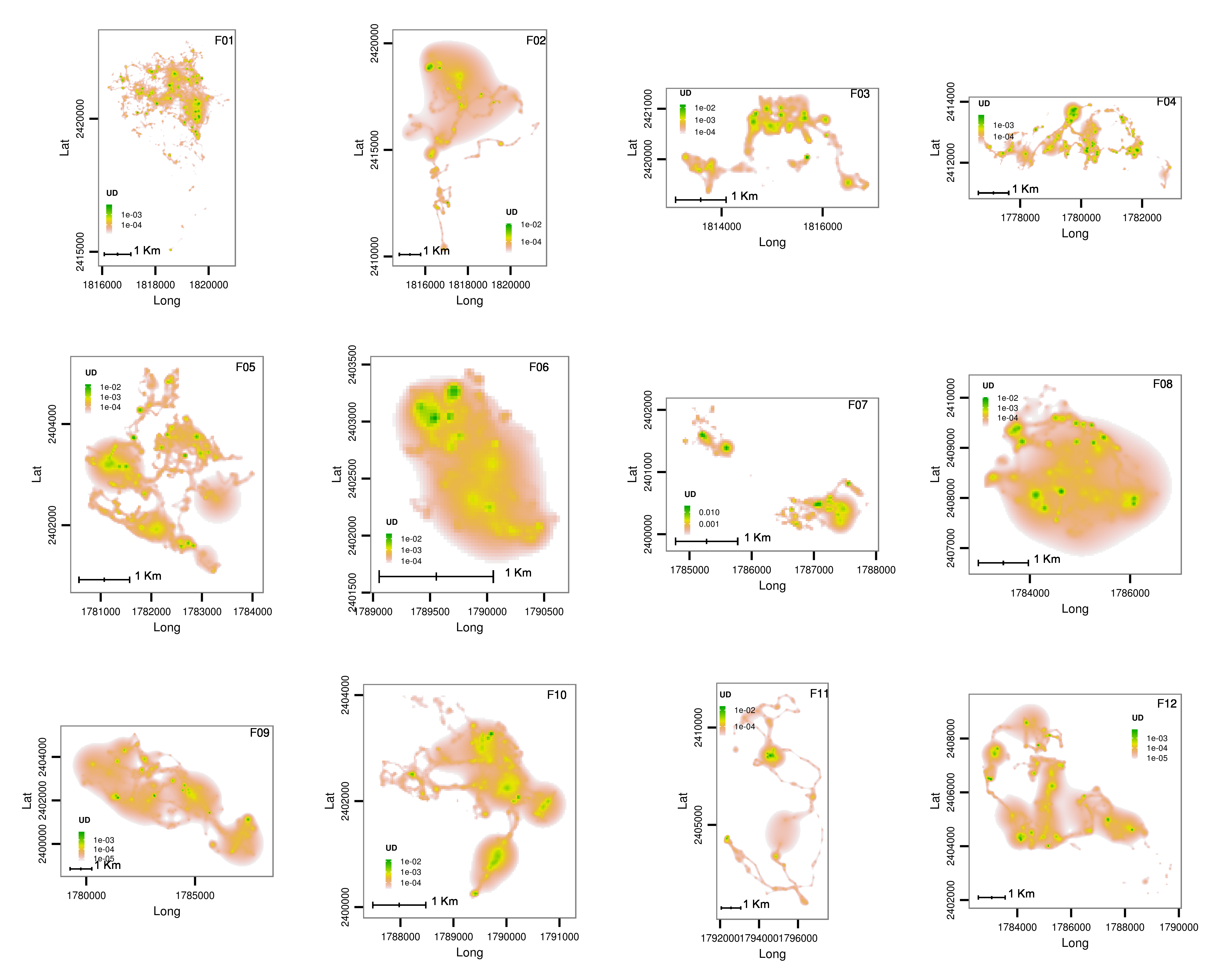

Supplement: S2 Fig — The color scale represents the relative proportion of time spent in each cell. The areas of the maps correspond to the home ranges of the individuals (95%UD). (TIF) [file pone.0145732.s003.tif]

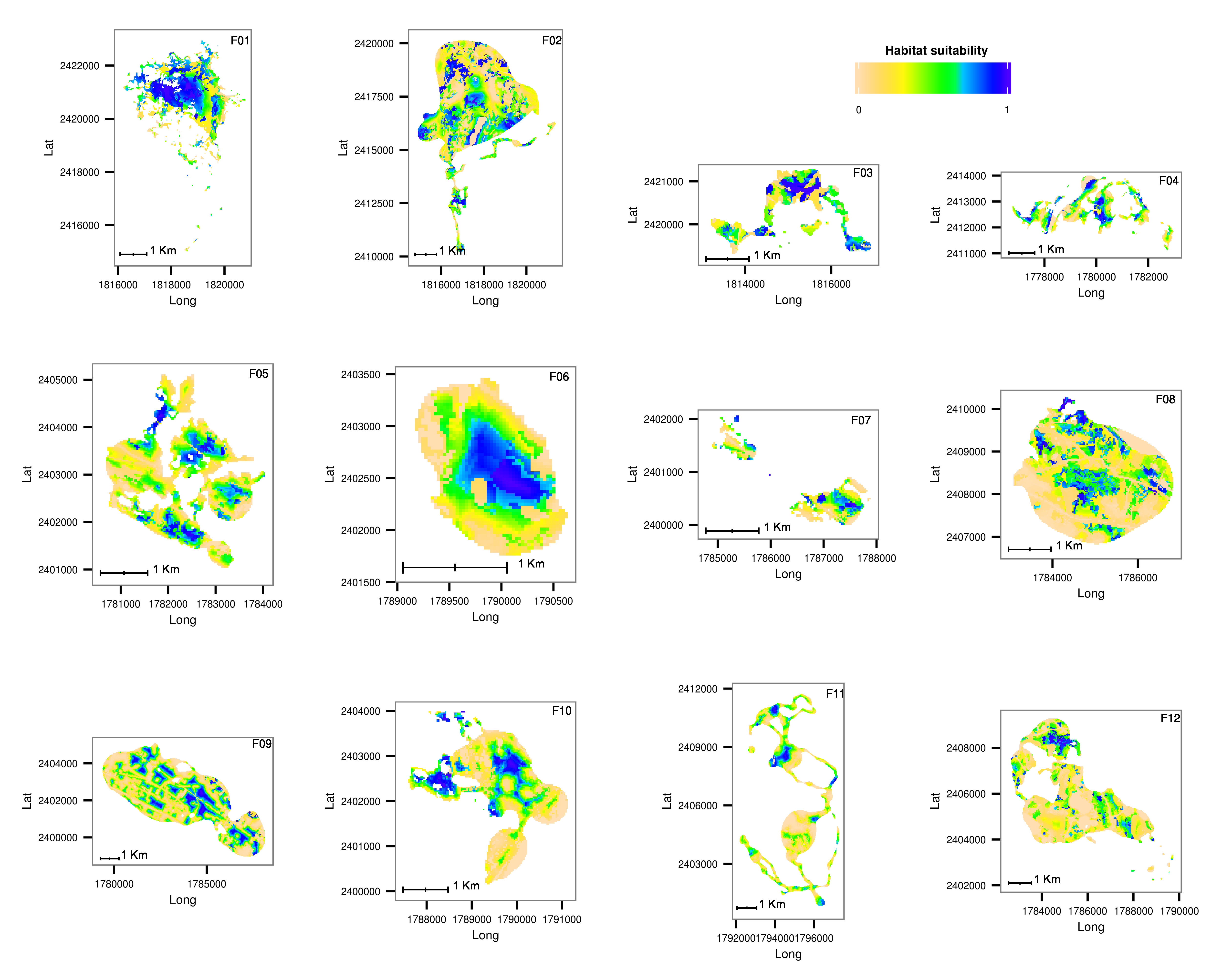

Supplement: S3 Fig — The areas of the maps correspond to the home ranges of the individuals (95%UD). (TIF) [file pone.0145732.s004.tif]
