# Supplementary material for: Acceleration Data Reveal Highly Individually Structured Energetic Landscapes in Free-Ranging Fishers (Pekania pennanti)
Source: PLoS One. 2016 Feb 3;11(2):e0145732. doi: 10.1371/journal.pone.0145732 (PMC4739643; doi:10.1371/journal.pone.0145732)
Supplement: S1 Table — (PDF) [file pone.0145732.s005.pdf]

S1 Table. Supplementary information of the tracked individuals

Individuals	Total number of points collected	GPS fix rate	Accelerometer settings
F01	17600	Dynamic sampling*	High sensitivity
F02	1077	10 min	High sensitivity
F03	874	10 min	High sensitivity
F04	10429	Dynamic sampling*	High sensitivity
F05	3444	Dynamic sampling*	High sensitivity
F06	1541	Dynamic sampling*	High sensitivity
F07	2824	Dynamic sampling*	High sensitivity
F08	1349	10 min	Low sensitivity
F09	919	15 min	High sensitivity
F10	2616	Dynamic sampling*	High sensitivity
F11	748	10 min	Low sensitivity
F12	1638	10 min	Low sensitivity

*GPS fixes every two minutes when the animal was highly active (e.g., running), every 10 minutes at moderate activity, and every 60 minutes during low activity (e.g., resting) (Brown et al. 2012)

References

Brown DD, LaPoint SD, Kays R, Heidrich W, Kümmeth F, Wikelski M. Accelerometer-informed GPS telemetry: Reducing the trade-off between resolution and longevity. Wildl Soc Bull. 2012;36: 139–146.
