# Supplementary material for: Acceleration Data Reveal Highly Individually Structured Energetic Landscapes in Free-Ranging Fishers (Pekania pennanti)
Source: PLoS One. 2016 Feb 3;11(2):e0145732. doi: 10.1371/journal.pone.0145732 (PMC4739643; doi:10.1371/journal.pone.0145732)
Supplement: S2 Table — (PDF) [file pone.0145732.s006.pdf]

S2 Table. Environmental variables included in the analyses

Variable	Description
Land cover:	
- developed low	Developed open space and developed low intensity*
- developed high	Developed medium intensity and developed high intensity*
- deciduous forest	Deciduous forest*
- evergreen forest	Evergreen forest*
- mixed forest	Mixed forest*
- shrub	Shrub/scrub*
- grassland	Grassland/herbaceous*
- crop	Pasture/ hay and cultivated crops*
- woody wetland	Woody wetland*
- herbaceous wetland	Emergent Herbaceous Wetlands*
- barren	Barren land*
- open water	Open water*
Distance to the forest edge (m)	Distance to deciduous,evergreen and mixed forest*. Positive values from inside the forest, and negative values form outside of the forest
Proportion of urban area (%)	% of developed high, medium and low intensity* within a 240 m radius circle from each 30m grid cell
Landscape heterogeneity	Number of different land cover types within a 240 m radius circle from each 30m grid cell
Distance to roads (m)	Distance from each grid cell to highways and paved roads#

*Classes correspond to the classification of the National Land Cover Database 2011 legend (Jin et al. 2011, NLCD11)

#Classification as provided by the United State Census Bureau (2011)

References

Jin S, Yang L, Danielson P, Homer C, Fry J, Xian G. A comprehensive change detection method for updating the National Land Cover Database to circa 2011. Remote Sens Environ. 2013;132: 159–175.

NLCD11. National Land Cover Database 2011. Available: http://www.mrlc.gov/nlcd2011.php

United States Census Bureau. TIGER/Line® Shapefiles and TIGER/Line® Files. 2011; Available: https://www.census.gov/geo/maps-data/data/tiger-line.html
