# Supplementary material for: Acceleration Data Reveal Highly Individually Structured Energetic Landscapes in Free-Ranging Fishers (Pekania pennanti)
Source: PLoS One. 2016 Feb 3;11(2):e0145732. doi: 10.1371/journal.pone.0145732 (PMC4739643; doi:10.1371/journal.pone.0145732)
Supplement: S3 Table — For each individual all possible combinations of the environmental variables were tested. Within each individual, the models are sorted by increasing ΔAIC values. (PDF) [file pone.0145732.s007.pdf]

**S3 Table. AICc values of GAMs including environmental variables.** For each individual all possible combinations of the environmental variables were tested. Within each individual, the models are sorted by increasing ΔAIC values.

| Intercept | Dist. Forest edge | Dist. Roads | Land cover | *Proportion developed* | *Landscape heterogeneity* | s(Space) | s(Time of day) | AICc | ΔAIC | Indiv. |
|---|---|---|---|---|---|---|---|---|---|---|
| 0.51553277 | *NA* | *NA* | *NA* | *NA* | -0.0168413891 | + | + | -1715.2139 | 0.000000000 | F1 |
| 0.51553069 | *NA* | *NA* | *NA* | 0.000000000 | -0.0168408305 | + | + | -1715.2074 | 0.006522108 | F1 |
| 0.48359502 | *NA* | 4.777449e-05 | *NA* | *NA* | -0.0162818549 | + | + | -1714.4327 | 0.781264177 | F1 |
| 0.48360045 | *NA* | 4.776292e-05 | *NA* | 0.000000000 | -0.0162813735 | + | + | -1714.4263 | 0.787677786 | F1 |
| 0.53503361 | *NA* | *NA* | + | *NA* | -0.0188670597 | + | + | -1714.1481 | 1.065837483 | F1 |
| 0.53503362 | *NA* | *NA* | + | 0.000000000 | -0.0188662115 | + | + | -1714.1407 | 1.073215573 | F1 |
| 0.51729367 | -3.410885e-06 | *NA* | *NA* | *NA* | -0.0169310722 | + | + | -1713.8841 | 1.329842608 | F1 |
| 0.51728584 | -3.399514e-06 | *NA* | *NA* | 0.000000000 | -0.0169302459 | + | + | -1713.8777 | 1.336238271 | F1 |
| 0.48912749 | -2.339468e-05 | 5.734013e-05 | *NA* | *NA* | -0.0167445104 | + | + | -1713.3885 | 1.825436075 | F1 |
| 0.48912884 | -2.337667e-05 | 5.732112e-05 | *NA* | 0.000000000 | -0.0167437071 | + | + | -1713.3821 | 1.831812276 | F1 |
| 0.52450587 | *NA* | 1.823139e-05 | + | *NA* | -0.0186535811 | + | + | -1713.0764 | 2.137561531 | F1 |
| 0.52451131 | *NA* | 1.822200e-05 | + | 0.000000000 | -0.0186528313 | + | + | -1713.0692 | 2.144764222 | F1 |
| 0.53908472 | -9.758053e-06 | *NA* | + | *NA* | -0.0191209465 | + | + | -1712.9761 | 2.237844810 | F1 |
| 0.53907741 | -9.740387e-06 | *NA* | + | 0.000000000 | -0.0191196812 | + | + | -1712.9687 | 2.245186403 | F1 |
| 0.52802537 | -1.889631e-05 | 2.572692e-05 | + | *NA* | -0.0190417051 | + | + | -1712.0826 | 3.131350291 | F1 |
| 0.52802632 | -1.887222e-05 | 2.570804e-05 | + | 0.000000000 | -0.0190405012 | + | + | -1712.0753 | 3.138637201 | F1 |
| 0.45284235 | *NA* | *NA* | *NA* | *NA* | *NA* | + | + | -1711.4561 | 3.757850744 | F1 |
| 0.45284235 | *NA* | *NA* | *NA* | 0.000000000 | *NA* | + | + | -1711.4495 | 3.764401244 | F1 |
| 0.40090757 | *NA* | 8.310711e-05 | *NA* | *NA* | *NA* | + | + | -1711.2602 | 3.953732210 | F1 |
| 0.40091526 | *NA* | 8.309480e-05 | *NA* | 0.000000000 | *NA* | + | + | -1711.2537 | 3.960195554 | F1 |
| 0.42896110 | 5.707965e-05 | *NA* | *NA* | *NA* | *NA* | + | + | -1710.0455 | 5.168447340 | F1 |
| 0.42895814 | 5.708672e-05 | *NA* | *NA* | 0.000000000 | *NA* | + | + | -1710.0391 | 5.174872334 | F1 |
| 0.39623571 | 3.234982e-05 | 6.892466e-05 | *NA* | *NA* | *NA* | + | + | -1709.7141 | 5.499821546 | F1 |
| 0.39624133 | 3.236326e-05 | 6.890666e-05 | *NA* | 0.000000000 | *NA* | + | + | -1709.7077 | 5.506204365 | F1 |
| 0.45600622 | *NA* | *NA* | + | *NA* | *NA* | + | + | -1708.5377 | 6.676256900 | F1 |
| 0.45600900 | *NA* | *NA* | + | 0.000000000 | *NA* | + | + | -1708.5302 | 6.683704028 | F1 |
| 0.42444484 | *NA* | 6.005194e-05 | + | *NA* | *NA* | + | + | -1707.9852 | 7.228692605 | F1 |
| 0.42445385 | *NA* | 6.004076e-05 | + | 0.000000000 | *NA* | + | + | -1707.9779 | 7.235991959 | F1 |
| 0.43709540 | 6.063262e-05 | *NA* | + | *NA* | *NA* | + | + | -1707.0042 | 8.209710406 | F1 |
| 0.43709518 | 6.064345e-05 | *NA* | + | 0.000000000 | *NA* | + | + | -1706.9968 | 8.217105817 | F1 |
| 0.42044710 | 4.607285e-05 | 4.025691e-05 | + | *NA* | *NA* | + | + | -1706.3114 | 8.902539298 | F1 |
| 0.42045467 | 4.609013e-05 | 4.023852e-05 | + | 0.000000000 | *NA* | + | + | -1706.3041 | 8.909859807 | F1 |
| 0.49248800 | *NA* | *NA* | *NA* | *NA* | *NA* | + | + | -356.0058 | 0.000000000 | F2 |
| 0.49248800 | *NA* | *NA* | *NA* | 0.000000000 | *NA* | + | + | -355.9330 | 0.072719813 | F2 |
| 0.49289646 | *NA* | -1.030512e-06 | *NA* | *NA* | *NA* | + | + | -355.3288 | 0.676915316 | F2 |
| 0.49283171 | *NA* | -8.671629e-07 | *NA* | 0.000000000 | *NA* | + | + | -355.2579 | 0.747890747 | F2 |
| 0.48203118 | 6.280356e-05 | *NA* | *NA* | *NA* | *NA* | + | + | -354.9700 | 1.035756697 | F2 |
| 0.48202675 | 6.283014e-05 | *NA* | *NA* | 0.000000000 | *NA* | + | + | -354.8980 | 1.107782947 | F2 |
| 0.49189161 | 7.230696e-05 | -2.886912e-05 | *NA* | *NA* | *NA* | + | + | -354.5034 | 1.502386811 | F2 |
| 0.49182549 | 7.227270e-05 | -2.868790e-05 | *NA* | 0.000000000 | *NA* | + | + | -354.4334 | 1.572331458 | F2 |
| 0.49905033 | *NA* | *NA* | *NA* | *NA* | -0.0015564940 | + | + | -354.2701 | 1.735680153 | F2 |
| 0.49907143 | *NA* | *NA* | *NA* | 0.000000000 | -0.0015614986 | + | + | -354.1983 | 1.807477651 | F2 |
| 0.50171857 | *NA* | -5.764143e-06 | *NA* | *NA* | -0.0016474591 | + | + | -353.5803 | 2.425491185 | F2 |
| 0.50166289 | *NA* | -5.605048e-06 | *NA* | 0.000000000 | -0.0016492113 | + | + | -353.5105 | 2.495290149 | F2 |
| 0.47707743 | 6.734442e-05 | *NA* | *NA* | *NA* | 0.0009956334 | + | + | -353.1154 | 2.890325830 | F2 |
| 0.47708251 | 6.736213e-05 | *NA* | *NA* | 0.000000000 | 0.0009937285 | + | + | -353.0450 | 2.960740709 | F2 |
| 0.48790312 | 7.551523e-05 | -2.818828e-05 | *NA* | *NA* | 0.0007553064 | + | + | -352.6212 | 3.384518415 | F2 |
| 0.48783958 | 7.547904e-05 | -2.800800e-05 | *NA* | 0.000000000 | 0.0007548597 | + | + | -352.5529 | 3.452900041 | F2 |
| 0.44017942 | *NA* | *NA* | + | *NA* | *NA* | + | + | -345.8715 | 10.134247013 | F2 |
| 0.44011930 | *NA* | *NA* | + | 0.000000000 | *NA* | + | + | -345.7527 | 10.253062043 | F2 |
| 0.43108378 | *NA* | 2.734067e-05 | + | *NA* | *NA* | + | + | -344.8989 | 11.106824202 | F2 |
| 0.43093564 | *NA* | 2.760727e-05 | + | 0.000000000 | *NA* | + | + | -344.7776 | 11.228190959 | F2 |
| 0.43080689 | 1.082037e-04 | *NA* | + | *NA* | *NA* | + | + | -343.7631 | 12.242652248 | F2 |
| 0.43074471 | 1.083683e-04 | *NA* | + | 0.000000000 | *NA* | + | + | -343.6319 | 12.373835898 | F2 |
| 0.46593063 | *NA* | *NA* | + | *NA* | -0.0073242672 | + | + | -343.6235 | 12.382257471 | F2 |

| Intercept | Dist. Forest edge | Dist. Roads | Land cover | *Proportion developed* | *Landscape heterogeneity* | s(Space) | s(Time of day) | AICc | ΔAIC | Indiv. |
|---|---|---|---|---|---|---|---|---|---|---|
| 0.43495045 | 1.115031e-04 | -1.236271e-05 | + | NA | NA | + | + | -343.5575 | 12.448310645 | F2 |
| 0.46593243 | NA | NA | + | 0.000000000 | -0.0073403562 | + | + | -343.4975 | 12.508269865 | F2 |
| 0.43480539 | 1.115720e-04 | -1.210168e-05 | + | 0.000000000 | NA | + | + | -343.4290 | 12.576790031 | F2 |
| 0.46252673 | NA | 7.844801e-06 | + | NA | -0.0070486296 | + | + | -343.0477 | 12.958021599 | F2 |
| 0.46241713 | NA | 8.100435e-06 | + | 0.000000000 | -0.0070577877 | + | + | -342.9226 | 13.083161375 | F2 |
| 0.44413574 | 9.200211e-05 | NA | + | NA | -0.0033718110 | + | + | -342.0368 | 13.968961794 | F2 |
| 0.44410250 | 9.213457e-05 | NA | + | 0.000000000 | -0.0033790596 | + | + | -341.9066 | 14.099144471 | F2 |
| 0.45038998 | 9.615917e-05 | -1.711228e-05 | + | NA | -0.0035332863 | + | + | -341.8829 | 14.122884815 | F2 |
| 0.45026091 | 9.621226e-05 | -1.685301e-05 | + | 0.000000000 | -0.0035372338 | + | + | -341.7558 | 14.249969798 | F2 |
| 0.49377385 | NA | NA | NA | NA | NA | + | + | -330.0687 | 0.000000000 | F3 |
| 0.49377385 | NA | NA | NA | 0.000000000 | NA | + | + | -329.9572 | 0.111497165 | F3 |
| 0.48232160 | NA | NA | NA | NA | 0.0017794976 | + | + | -328.6224 | 1.446367096 | F3 |
| 0.48281114 | NA | NA | NA | 0.000000000 | 0.0017034310 | + | + | -328.4925 | 1.576174837 | F3 |
| 0.50566968 | -3.010230e-04 | NA | NA | NA | NA | + | + | -327.8575 | 2.211208316 | F3 |
| 0.50569272 | -3.016059e-04 | NA | NA | 0.000000000 | NA | + | + | -327.7398 | 2.328945690 | F3 |
| 0.45348035 | NA | 1.425371e-04 | NA | NA | NA | + | + | -327.5178 | 2.550927393 | F3 |
| 0.45306283 | NA | 1.440141e-04 | NA | 0.000000000 | NA | + | + | -327.3872 | 2.681550938 | F3 |
| 0.49275233 | -3.006511e-04 | NA | NA | NA | 0.0020048672 | + | + | -326.4926 | 3.576093429 | F3 |
| 0.43273135 | NA | 1.461145e-04 | NA | NA | 0.0030669267 | + | + | -326.4468 | 3.621885071 | F3 |
| 0.49325119 | -3.012041e-04 | NA | NA | 0.000000000 | 0.0019307480 | + | + | -326.3578 | 3.710951580 | F3 |
| 0.43277753 | NA | 1.474299e-04 | NA | 0.000000000 | 0.0030019745 | + | + | -326.3050 | 3.763699793 | F3 |
| 0.43676659 | -3.821071e-04 | 2.550779e-04 | NA | NA | NA | + | + | -322.4217 | 7.647026897 | F3 |
| 0.43629537 | -3.836484e-04 | 2.569602e-04 | NA | 0.000000000 | NA | + | + | -322.2558 | 7.812910263 | F3 |
| 0.41038814 | -3.830786e-04 | 2.611429e-04 | NA | NA | 0.0038383472 | + | + | -321.6293 | 8.439393833 | F3 |
| 0.41035644 | -3.845405e-04 | 2.628663e-04 | NA | 0.000000000 | 0.0037765468 | + | + | -321.4538 | 8.614888660 | F3 |
| 0.57871822 | NA | NA | + | NA | NA | + | + | -314.0859 | 15.982790647 | F3 |
| 0.57870616 | NA | NA | + | 0.000000000 | NA | + | + | -313.9515 | 16.117232119 | F3 |
| 0.56800644 | NA | NA | + | NA | 0.0017797970 | + | + | -312.3266 | 17.742162562 | F3 |
| 0.56866617 | NA | NA | + | 0.000000000 | 0.0016715115 | + | + | -312.1530 | 17.915698745 | F3 |
| 0.57456044 | -2.720802e-04 | NA | + | NA | NA | + | + | -312.0791 | 17.989610339 | F3 |
| 0.57449107 | -2.726798e-04 | NA | + | 0.000000000 | NA | + | + | -311.9436 | 18.125157250 | F3 |
| 0.54045871 | NA | 1.621540e-04 | + | NA | NA | + | + | -310.9076 | 19.161141142 | F3 |
| 0.54008242 | NA | 1.638106e-04 | + | 0.000000000 | NA | + | + | -310.7474 | 19.321356920 | F3 |
| 0.56248671 | -2.715982e-04 | NA | + | NA | 0.0020479529 | + | + | -310.4014 | 19.667335315 | F3 |
| 0.56308628 | -2.721509e-04 | NA | + | 0.000000000 | 0.0019396504 | + | + | -310.2272 | 19.841510528 | F3 |
| 0.51941701 | NA | 1.660743e-04 | + | NA | 0.0033076556 | + | + | -309.7253 | 20.343400099 | F3 |
| 0.51968727 | NA | 1.675122e-04 | + | 0.000000000 | 0.0032136054 | + | + | -309.5371 | 20.531580445 | F3 |
| 0.51579653 | -3.518147e-04 | 2.413731e-04 | + | NA | NA | + | + | -307.1044 | 22.964295800 | F3 |
| 0.51529195 | -3.533036e-04 | 2.431990e-04 | + | 0.000000000 | NA | + | + | -306.9348 | 23.133966503 | F3 |
| 0.49067106 | -3.529216e-04 | 2.480222e-04 | + | NA | 0.0039601114 | + | + | -306.1325 | 23.936175466 | F3 |
| 0.49081110 | -3.543023e-04 | 2.496283e-04 | + | 0.000000000 | 0.0038665739 | + | + | -305.9345 | 24.134244475 | F3 |
| 0.39005716 | -6.181833e-04 | NA | + | NA | 0.0127404134 | + | + | -1016.2407 | 0.000000000 | F4 |
| 0.39047359 | -6.186046e-04 | -2.387918e-06 | + | NA | 0.0127577191 | + | + | -1015.1909 | 1.049746562 | F4 |
| 0.39017211 | -6.227061e-04 | NA | + | -0.138788455 | 0.0126856015 | + | + | -1015.0519 | 1.188814353 | F4 |
| 0.39133631 | -6.230746e-04 | -4.977568e-06 | + | -0.141453158 | 0.0126874030 | + | + | -1013.9725 | 2.268134672 | F4 |
| 0.48003282 | -6.119177e-04 | NA | + | NA | NA | + | + | -1010.0250 | 6.215640920 | F4 |
| 0.47961500 | -6.188066e-04 | NA | + | -0.218016416 | NA | + | + | -1009.0108 | 7.229909895 | F4 |
| 0.49205406 | -6.102630e-04 | -4.880018e-05 | + | NA | NA | + | + | -1008.8859 | 7.354757618 | F4 |
| 0.49260700 | -6.174601e-04 | -5.282135e-05 | + | -0.234620970 | NA | + | + | -1007.8559 | 8.384807678 | F4 |
| 0.40159679 | NA | NA | + | NA | 0.0125110209 | + | + | -1005.7816 | 10.459111428 | F4 |
| 0.40711873 | NA | -2.030666e-05 | + | NA | 0.0124307062 | + | + | -1004.6904 | 11.550242000 | F4 |
| 0.40125514 | NA | NA | + | 0.064630351 | 0.0125651988 | + | + | -1004.3932 | 11.847503361 | F4 |
| 0.40639893 | NA | -1.889533e-05 | + | 0.057407727 | 0.0124879666 | + | + | -1003.2931 | 12.947541661 | F4 |
| 0.48986714 | NA | NA | + | NA | NA | + | + | -1000.3415 | 15.899182156 | F4 |
| 0.50584574 | NA | -6.497009e-05 | + | NA | NA | + | + | -999.2292 | 17.011438979 | F4 |
| 0.48980033 | NA | NA | + | -0.016428462 | NA | + | + | -999.0325 | 17.208136522 | F4 |
| 0.50588673 | NA | -6.550467e-05 | + | -0.037404003 | NA | + | + | -997.9211 | 18.319615139 | F4 |
| 0.38937821 | -4.613329e-04 | NA | NA | NA | 0.0096775859 | + | + | -993.0797 | 23.160920664 | F4 |
| 0.39730530 | -4.604336e-04 | -2.983383e-05 | NA | NA | 0.0095424401 | + | + | -991.9016 | 24.339079878 | F4 |
| 0.38984638 | -4.631040e-04 | NA | NA | -0.079162374 | 0.0096532124 | + | + | -991.8010 | 24.439672107 | F4 |
| 0.39830142 | -4.623645e-04 | -3.145973e-05 | NA | -0.090560430 | 0.0095029979 | + | + | -990.6000 | 25.640653910 | F4 |
| 0.46046617 | -4.599929e-04 | NA | NA | NA | NA | + | + | -989.7636 | 26.477103709 | F4 |
| 0.46096650 | -4.629549e-04 | NA | NA | -0.139141318 | NA | + | + | -988.5784 | 27.662299519 | F4 |

| Intercept | Dist. Forest edge | Dist. Roads | Land cover | *Proportion developed* | *Landscape heterogeneity* | s(Space) | s(Time of day) | AICc | ΔAIC | Indiv. |
|---|---|---|---|---|---|---|---|---|---|---|
| 0.47574310 | -4.577351e-04 | -6.579430e-05 | NA | NA | NA | + | + | -988.5325 | 27.708199787 | F4 |
| 0.47697612 | -4.610341e-04 | -6.861588e-05 | NA | -0.161200026 | NA | + | + | -987.3312 | 28.909424340 | F4 |
| 0.36649172 | NA | NA | NA | NA | 0.0096467803 | + | + | -987.0308 | 29.209888056 | F4 |
| 0.37840737 | NA | -4.422523e-05 | NA | NA | 0.0094331337 | + | + | -985.8266 | 30.414031661 | F4 |
| 0.36602954 | NA | NA | NA | 0.053777884 | 0.0096911405 | + | + | -985.6678 | 30.572832231 | F4 |
| 0.37774730 | NA | -4.313188e-05 | NA | 0.038020016 | 0.0094750452 | + | + | -984.4600 | 31.780626162 | F4 |
| 0.43742032 | NA | NA | NA | NA | NA | + | + | -984.1363 | 32.104392120 | F4 |
| 0.45597517 | NA | -7.932421e-05 | NA | NA | NA | + | + | -982.9191 | 33.321544010 | F4 |
| 0.43743762 | NA | NA | NA | -0.006840523 | NA | + | + | -982.8225 | 33.418127566 | F4 |
| 0.45615587 | NA | -7.974041e-05 | NA | -0.032949065 | NA | + | + | -981.6072 | 34.633447572 | F4 |
| 0.28738749 | -3.954497e-04 | NA | + | NA | -0.0365019473 | + | + | -363.8175 | 0.000000000 | F5 |
| 0.28718229 | -3.926321e-04 | NA | + | -2.978949450 | -0.0364552028 | + | + | -362.3850 | 1.432522192 | F5 |
| 0.26646501 | NA | NA | + | NA | -0.0322884776 | + | + | -362.0176 | 1.799877933 | F5 |
| 0.26639697 | NA | NA | + | -3.387832627 | -0.0322838333 | + | + | -360.7394 | 3.078058302 | F5 |
| 0.32613455 | -3.501629e-04 | -1.728894e-04 | + | NA | -0.0376815000 | + | + | -360.5721 | 3.245391700 | F5 |
| 0.32554951 | -3.480168e-04 | -1.712626e-04 | + | -2.913215691 | -0.0376198760 | + | + | -359.1656 | 4.651878457 | F5 |
| 0.32243322 | NA | -2.347086e-04 | + | NA | -0.0345040487 | + | + | -358.4990 | 5.318525854 | F5 |
| 0.32178935 | NA | -2.324801e-04 | + | -3.209861914 | -0.0344667067 | + | + | -357.2328 | 6.584655127 | F5 |
| 0.09968288 | -2.133755e-04 | NA | + | NA | NA | + | + | -355.5171 | 8.300386804 | F5 |
| 0.10056211 | NA | NA | + | NA | NA | + | + | -355.1651 | 8.652426057 | F5 |
| 0.09971542 | -2.108269e-04 | NA | + | -3.387669976 | NA | + | + | -354.1788 | 9.638637827 | F5 |
| 0.10053325 | NA | NA | + | -3.563146675 | NA | + | + | -353.9225 | 9.894974292 | F5 |
| 0.11409173 | -1.910559e-04 | -7.743858e-05 | + | NA | NA | + | + | -353.4842 | 10.333249559 | F5 |
| 0.12249125 | NA | -1.175536e-04 | + | NA | NA | + | + | -353.0155 | 10.802015983 | F5 |
| 0.11389555 | -1.889582e-04 | -7.618979e-05 | + | -3.384681038 | NA | + | + | -352.1589 | 11.658621713 | F5 |
| 0.12214155 | NA | -1.157616e-04 | + | -3.513824134 | NA | + | + | -351.7789 | 12.038586085 | F5 |
| 0.70799644 | NA | NA | NA | NA | -0.0292499341 | + | + | -344.9148 | 18.902672875 | F5 |
| 0.70794458 | NA | NA | NA | -4.697389186 | -0.0291977117 | + | + | -344.1242 | 19.693296935 | F5 |
| 0.73429458 | -2.127589e-04 | NA | NA | NA | -0.0312272291 | + | + | -344.1204 | 19.697059754 | F5 |
| 0.73466544 | -2.169335e-04 | NA | NA | -4.916261056 | -0.0311956094 | + | + | -343.3492 | 20.468324493 | F5 |
| 0.73860982 | NA | -1.196636e-04 | NA | NA | -0.0304817079 | + | + | -342.3762 | 21.441318171 | F5 |
| 0.53935279 | NA | NA | NA | NA | NA | + | + | -342.0037 | 21.813823682 | F5 |
| 0.75256517 | -1.940201e-04 | -8.029117e-05 | NA | NA | -0.0318875483 | + | + | -341.9518 | 21.865690840 | F5 |
| 0.73830488 | NA | -1.189217e-04 | NA | -4.705054030 | -0.0304108021 | + | + | -341.5995 | 22.217972309 | F5 |
| 0.53961571 | NA | NA | NA | -4.955364834 | NA | + | + | -341.2378 | 22.579687190 | F5 |
| 0.75255664 | -1.986263e-04 | -7.873057e-05 | NA | -4.916599906 | -0.0318380624 | + | + | -341.1994 | 22.618052088 | F5 |
| 0.54653753 | -1.026067e-04 | NA | NA | NA | NA | + | + | -341.0714 | 22.746119912 | F5 |
| 0.54180378 | NA | -1.247456e-05 | NA | NA | NA | + | + | -340.7948 | 23.022638079 | F5 |
| 0.54715870 | -1.076147e-04 | NA | NA | -5.097982283 | NA | + | + | -340.3247 | 23.492831686 | F5 |
| 0.54434892 | -1.065648e-04 | 1.254971e-05 | NA | NA | NA | + | + | -340.0428 | 23.774678059 | F5 |
| 0.54208567 | NA | -1.256233e-05 | NA | -4.987603752 | NA | + | + | -340.0204 | 23.797075218 | F5 |
| 0.54479042 | -1.118207e-04 | 1.356102e-05 | NA | -5.129612421 | NA | + | + | -339.2972 | 24.520262278 | F5 |
| 0.53573599 | NA | 2.264863e-04 | NA | NA | NA | + | + | -291.2640 | 0.00000000 | F6 |
| 0.54053281 | NA | 2.204332e-04 | NA | -0.225809767 | NA | + | + | -290.8624 | 0.40152922 | F6 |
| 0.59074183 | NA | NA | NA | NA | NA | + | + | -290.4113 | 0.85263178 | F6 |
| 0.59590496 | NA | NA | NA | -0.350459334 | NA | + | + | -290.3574 | 0.90655227 | F6 |
| 0.53583439 | -2.862122e-05 | 2.363337e-04 | NA | NA | NA | + | + | -290.0278 | 1.23612518 | F6 |
| 0.54050216 | -2.726377e-05 | 2.303237e-04 | NA | -0.225776849 | NA | + | + | -289.6068 | 1.65715607 | F6 |
| 0.59195872 | -1.398752e-05 | NA | NA | NA | NA | + | + | -289.2447 | 2.01922231 | F6 |
| 0.59728392 | -1.526501e-05 | NA | NA | -0.353916779 | NA | + | + | -289.1482 | 2.11571007 | F6 |
| 0.55170497 | NA | 2.256218e-04 | + | NA | NA | + | + | -284.0541 | 7.20986072 | F6 |
| 0.57474355 | NA | 1.986981e-04 | + | -0.474212330 | NA | + | + | -283.6607 | 7.60320798 | F6 |
| 0.63343869 | NA | NA | + | -0.595784412 | NA | + | + | -283.3761 | 7.88789257 | F6 |
| 0.61266703 | NA | NA | + | NA | NA | + | + | -283.3272 | 7.93672470 | F6 |
| 0.54661895 | 7.640010e-05 | 2.255906e-04 | + | NA | NA | + | + | -283.1260 | 8.13792760 | F6 |
| 0.56882563 | 7.637169e-05 | 1.994871e-04 | + | -0.464781626 | NA | + | + | -282.6618 | 8.60217485 | F6 |
| 0.60684985 | 9.114338e-05 | NA | + | NA | NA | + | + | -282.5638 | 8.70015956 | F6 |
| 0.62708663 | 8.724359e-05 | NA | + | -0.586062424 | NA | + | + | -282.4929 | 8.77107119 | F6 |
| 0.43049785 | NA | NA | NA | -0.331507359 | 0.0344933845 | + | + | -282.0715 | 9.19240844 | F6 |
| 0.42470905 | NA | NA | NA | NA | 0.0346824018 | + | + | -281.9316 | 9.33232783 | F6 |
| 0.43267575 | -3.630496e-06 | NA | NA | -0.328264428 | 0.0340944405 | + | + | -281.5473 | 9.71666074 | F6 |
| 0.42590215 | 2.678288e-06 | NA | NA | NA | 0.0343845049 | + | + | -281.3902 | 9.87377987 | F6 |
| 0.30583209 | NA | 3.906569e-04 | NA | -0.041696924 | 0.0398239832 | + | + | -279.9208 | 11.34312180 | F6 |

| Intercept | Dist. Forest edge | Dist. Roads | Land cover | *Proportion developed* | *Landscape heterogeneity* | s(Space) | s(Time of day) | AICc | ΔAIC | Indiv. |
|---|---|---|---|---|---|---|---|---|---|---|
| 0.31119267 | -4.099861e-05 | 3.975495e-04 | *NA* | -0.044424401 | 0.0391079982 | + | + | -279.7244 | 11.53952497 | F6 |
| 0.30097994 | *NA* | 3.908771e-04 | *NA* | *NA* | 0.0406980502 | + | + | -279.2984 | 11.96557846 | F6 |
| 0.30612653 | -3.891804e-05 | 3.976016e-04 | *NA* | *NA* | 0.0399890930 | + | + | -279.1210 | 12.14295747 | F6 |
| 0.48471381 | *NA* | *NA* | + | *NA* | 0.0373246994 | + | + | -274.5927 | 16.67124664 | F6 |
| 0.47608090 | 1.226857e-04 | *NA* | + | *NA* | 0.0379048278 | + | + | -273.5317 | 17.73227044 | F6 |
| 0.35227534 | *NA* | 4.180405e-04 | + | *NA* | 0.0450519270 | + | + | -271.4587 | 19.80527039 | F6 |
| 0.51975981 | *NA* | *NA* | + | -0.733047996 | 0.0387492838 | + | + | -271.3103 | 19.95361187 | F6 |
| 0.50984333 | 1.211902e-04 | *NA* | + | -0.720260903 | 0.0392878669 | + | + | -270.1367 | 21.12722389 | F6 |
| 0.34812531 | 8.336899e-05 | 4.122307e-04 | + | *NA* | 0.0453350752 | + | + | -269.9868 | 21.27717343 | F6 |
| 0.38880598 | *NA* | 3.767122e-04 | + | -0.442298823 | 0.0452273982 | + | + | -269.0214 | 22.24253594 | F6 |
| 0.38366747 | 8.585607e-05 | 3.713147e-04 | + | -0.437116831 | 0.0453706483 | + | + | -267.4551 | 23.80889861 | F6 |
| 0.69677993 | *NA* | -6.962629e-04 | *NA* | -2.964730404 | *NA* | + | + | -535.3741 | 0.00000000 | F7 |
| 0.68647971 | 1.402339e-04 | -6.904601e-04 | *NA* | -3.082125469 | *NA* | + | + | -535.2072 | 0.16688200 | F7 |
| 0.73055593 | *NA* | -7.126672e-04 | *NA* | -2.970593616 | -0.0051961168 | + | + | -534.1199 | 1.25417851 | F7 |
| 0.72124807 | 1.437931e-04 | -7.066894e-04 | *NA* | -3.089925372 | -0.0054116653 | + | + | -533.9198 | 1.45430938 | F7 |
| 0.68664110 | *NA* | -6.654918e-04 | *NA* | *NA* | *NA* | + | + | -532.9676 | 2.40646388 | F7 |
| 0.67905986 | 1.050934e-04 | -6.623630e-04 | *NA* | *NA* | *NA* | + | + | -532.7395 | 2.63459704 | F7 |
| 0.72152279 | *NA* | -6.815654e-04 | *NA* | *NA* | -0.0053980575 | + | + | -531.6416 | 3.73252080 | F7 |
| 0.71450356 | 1.087820e-04 | -6.779978e-04 | *NA* | *NA* | -0.0055511988 | + | + | -531.3666 | 4.00751961 | F7 |
| 0.55331060 | *NA* | *NA* | *NA* | -2.766978759 | *NA* | + | + | -529.6568 | 5.71724176 | F7 |
| 0.54162660 | 1.797493e-04 | *NA* | *NA* | -2.908676763 | *NA* | + | + | -529.6354 | 5.73870027 | F7 |
| 0.56822159 | *NA* | *NA* | *NA* | -2.773094772 | -0.0025477993 | + | + | -528.3531 | 7.02101654 | F7 |
| 0.55860836 | 1.825541e-04 | *NA* | *NA* | -2.915033060 | -0.0029334121 | + | + | -528.3152 | 7.05883809 | F7 |
| 0.54975522 | *NA* | *NA* | *NA* | *NA* | *NA* | + | + | -527.9776 | 7.39651231 | F7 |
| 0.54015601 | 1.454106e-04 | *NA* | *NA* | *NA* | *NA* | + | + | -527.9042 | 7.46991714 | F7 |
| 0.56630425 | *NA* | *NA* | *NA* | *NA* | -0.0028291763 | + | + | -526.6712 | 8.70284907 | F7 |
| 0.55839454 | 1.484565e-04 | *NA* | *NA* | *NA* | -0.0031523834 | + | + | -526.5631 | 8.81100505 | F7 |
| 0.69686828 | *NA* | -6.674341e-04 | + | -3.045967458 | *NA* | + | + | -525.4252 | 9.94884609 | F7 |
| 0.69652066 | 1.672984e-04 | -6.638326e-04 | + | -3.206047699 | *NA* | + | + | -525.3362 | 10.03789328 | F7 |
| 0.72436338 | *NA* | -6.825299e-04 | + | -3.053231358 | -0.0040949552 | + | + | -524.1670 | 11.20702867 | F7 |
| 0.72635147 | 1.723766e-04 | -6.793331e-04 | + | -3.216584083 | -0.0044753628 | + | + | -524.0759 | 11.29813360 | F7 |
| 0.68465926 | *NA* | -6.365051e-04 | + | *NA* | *NA* | + | + | -523.0643 | 12.30975249 | F7 |
| 0.68473588 | 1.159305e-04 | -6.360972e-04 | + | *NA* | *NA* | + | + | -522.8989 | 12.47521018 | F7 |
| 0.71354919 | *NA* | -6.514784e-04 | + | *NA* | -0.0043389545 | + | + | -521.7624 | 13.61170477 | F7 |
| 0.71516151 | 1.210306e-04 | -6.510063e-04 | + | *NA* | -0.0045998086 | + | + | -521.5684 | 13.80564564 | F7 |
| 0.55182012 | 1.985586e-04 | *NA* | + | -3.035697900 | *NA* | + | + | -520.0936 | 15.28045800 | F7 |
| 0.55078539 | *NA* | *NA* | + | -2.848291140 | *NA* | + | + | -520.0223 | 15.35173391 | F7 |
| 0.56296907 | 2.016194e-04 | *NA* | + | -3.044617920 | -0.0018661704 | + | + | -518.7384 | 16.63569156 | F7 |
| 0.55873790 | *NA* | *NA* | + | -2.856924132 | -0.0013211804 | + | + | -518.6455 | 16.72858585 | F7 |
| 0.54522683 | *NA* | *NA* | + | *NA* | *NA* | + | + | -518.3267 | 17.04733753 | F7 |
| 0.54591314 | 1.483336e-04 | *NA* | + | *NA* | *NA* | + | + | -518.3133 | 17.06074519 | F7 |
| 0.55523025 | *NA* | *NA* | + | *NA* | -0.0016726164 | + | + | -516.9838 | 18.39024867 | F7 |
| 0.55840740 | 1.516542e-04 | *NA* | + | *NA* | -0.0020968343 | + | + | -516.9636 | 18.41045503 | F7 |
| 0.66974506 | *NA* | *NA* | + | *NA* | *NA* | + | + | -179.9039 | 0.00000000 | F8 |
| 0.66977373 | *NA* | *NA* | + | 0.000000000 | *NA* | + | + | -179.8510 | 0.05286103 | F8 |
| 0.66453342 | 2.481802e-04 | *NA* | + | *NA* | *NA* | + | + | -179.1405 | 0.76342113 | F8 |
| 0.66456868 | 2.480809e-04 | *NA* | + | 0.000000000 | *NA* | + | + | -179.0891 | 0.81482224 | F8 |
| 0.71889371 | *NA* | -2.736610e-04 | + | *NA* | *NA* | + | + | -178.4594 | 1.44449089 | F8 |
| 0.39159425 | *NA* | *NA* | *NA* | *NA* | *NA* | + | + | -178.4186 | 1.48527180 | F8 |
| 0.71895795 | *NA* | -2.738455e-04 | + | 0.000000000 | *NA* | + | + | -178.4076 | 1.49632858 | F8 |
| 0.39159425 | *NA* | *NA* | *NA* | 0.000000000 | *NA* | + | + | -178.3071 | 1.59684989 | F8 |
| 0.74669368 | 4.993309e-04 | -4.852330e-04 | + | *NA* | *NA* | + | + | -178.1025 | 1.80138368 | F8 |
| 0.72612542 | *NA* | *NA* | + | *NA* | -0.0073330583 | + | + | -178.0659 | 1.83803418 | F8 |
| 0.74676376 | 4.993259e-04 | -4.853813e-04 | + | 0.000000000 | *NA* | + | + | -178.0490 | 1.85488524 | F8 |
| 0.72620540 | *NA* | *NA* | + | 0.000000000 | -0.0073394542 | + | + | -178.0137 | 1.89022478 | F8 |
| 0.45627525 | *NA* | -3.324385e-04 | *NA* | *NA* | *NA* | + | + | -177.5056 | 2.39830751 | F8 |
| 0.45630945 | *NA* | -3.326143e-04 | *NA* | 0.000000000 | *NA* | + | + | -177.4070 | 2.49688125 | F8 |
| 0.70092653 | 2.220060e-04 | *NA* | + | *NA* | -0.0046661906 | + | + | -177.2238 | 2.68006583 | F8 |
| 0.70101088 | 2.218654e-04 | *NA* | + | 0.000000000 | -0.0046724202 | + | + | -177.1724 | 2.73148655 | F8 |
| 0.39068621 | *NA* | *NA* | *NA* | *NA* | 0.0001431974 | + | + | -176.5172 | 3.38667587 | F8 |
| 0.40064957 | -1.490782e-04 | *NA* | *NA* | *NA* | *NA* | + | + | -176.5000 | 3.40392866 | F8 |
| 0.80149417 | *NA* | -3.063919e-04 | + | *NA* | -0.0099696524 | + | + | -176.4700 | 3.43393036 | F8 |
| 0.80164923 | *NA* | -3.066144e-04 | + | 0.000000000 | -0.0099801242 | + | + | -176.4169 | 3.48703487 | F8 |

| Intercept | Dist. Forest edge | Dist. Roads | Land cover | *Proportion developed* | *Landscape heterogeneity* | s(Space) | s(Time of day) | AICc | ΔAIC | Indiv. |
|---|---|---|---|---|---|---|---|---|---|---|
| 0.39073782 | NA | NA | NA | 0.000000000 | 0.0001350580 | + | + | -176.4041 | 3.49978227 | F8 |
| 0.40065637 | -1.491900e-04 | NA | NA | 0.000000000 | NA | + | + | -176.3768 | 3.52712267 | F8 |
| 0.79399473 | 4.693683e-04 | -4.917590e-04 | + | NA | -0.0059187725 | + | + | -176.1528 | 3.75110993 | F8 |
| 0.79412912 | 4.693160e-04 | -4.919170e-04 | + | 0.000000000 | -0.0059266519 | + | + | -176.0997 | 3.80422955 | F8 |
| 0.45610494 | -2.058964e-06 | -3.309204e-04 | NA | NA | NA | + | + | -176.0111 | 3.89282357 | F8 |
| 0.45613846 | -2.073602e-06 | -3.310881e-04 | NA | 0.000000000 | NA | + | + | -175.9114 | 3.99251650 | F8 |
| 0.47746607 | NA | -3.430721e-04 | NA | NA | -0.0030155133 | + | + | -175.4222 | 4.48170330 | F8 |
| 0.47758873 | NA | -3.432933e-04 | NA | 0.000000000 | -0.0030280695 | + | + | -175.3226 | 4.58131198 | F8 |
| 0.41619702 | -1.626016e-04 | NA | NA | NA | -0.0023222829 | + | + | -174.3364 | 5.56747625 | F8 |
| 0.41628436 | -1.627845e-04 | NA | NA | 0.000000000 | -0.0023343042 | + | + | -174.2085 | 5.69540400 | F8 |
| 0.47811517 | -1.918009e-05 | -3.344350e-04 | NA | NA | -0.0031991604 | + | + | -173.8392 | 6.06473875 | F8 |
| 0.47824235 | -1.926705e-05 | -3.346196e-04 | NA | 0.000000000 | -0.0032127185 | + | + | -173.7371 | 6.16677706 | F8 |
| 0.49582597 | -5.556896e-04 | NA | NA | NA | NA | + | + | -172.0894 | 0.00000000 | F9 |
| 0.53986938 | -5.961111e-04 | NA | NA | NA | -0.0071771559 | + | + | -170.2452 | 1.84418559 | F9 |
| 0.54996567 | -4.976312e-04 | -2.771063e-04 | NA | NA | NA | + | + | -169.8915 | 2.19784310 | F9 |
| 0.50215794 | -5.665291e-04 | NA | NA | -0.325621126 | NA | + | + | -168.9017 | 3.18763074 | F9 |
| 0.59957034 | -5.417400e-04 | -2.815552e-04 | NA | NA | -0.0079383899 | + | + | -168.0730 | 4.01635596 | F9 |
| 0.54837046 | -6.092219e-04 | NA | NA | -0.341280358 | -0.0074805894 | + | + | -166.9873 | 5.10210176 | F9 |
| 0.55506035 | -5.085566e-04 | -2.730311e-04 | NA | -0.302983350 | NA | + | + | -166.8797 | 5.20968427 | F9 |
| 0.60656517 | -5.547654e-04 | -2.773457e-04 | NA | -0.319268093 | -0.0082001412 | + | + | -164.9945 | 7.09483740 | F9 |
| 0.66175837 | -4.764166e-04 | NA | + | NA | NA | + | + | -164.9465 | 7.14281505 | F9 |
| 0.46125787 | NA | NA | NA | NA | NA | + | + | -164.8744 | 7.21492780 | F9 |
| 0.53422762 | NA | -3.501281e-04 | NA | NA | NA | + | + | -164.4048 | 7.68456996 | F9 |
| 0.44446346 | NA | NA | NA | NA | 0.0029024643 | + | + | -163.5993 | 8.49009714 | F9 |
| 0.73129543 | -4.150145e-04 | -3.273969e-04 | + | NA | NA | + | + | -163.3008 | 8.78855633 | F9 |
| 0.68567926 | -5.101382e-04 | NA | + | NA | -0.0045933858 | + | + | -163.1095 | 8.97989780 | F9 |
| 0.52858376 | NA | -3.488156e-04 | NA | NA | 0.0009281169 | + | + | -162.9797 | 9.10960799 | F9 |
| 0.46361708 | NA | NA | NA | -0.135782042 | NA | + | + | -162.3001 | 9.78923351 | F9 |
| 0.53620065 | NA | -3.487838e-04 | NA | -0.129680218 | NA | + | + | -161.9391 | 10.15029601 | F9 |
| 0.75998126 | -4.546404e-04 | -3.299564e-04 | + | NA | -0.0054337715 | + | + | -161.4455 | 10.64384411 | F9 |
| 0.70470821 | -4.737896e-04 | NA | + | -0.664463368 | NA | + | + | -161.0685 | 11.02083529 | F9 |
| 0.44677671 | NA | NA | NA | -0.129874464 | 0.0028926684 | + | + | -161.0675 | 11.02185292 | F9 |
| 0.75047679 | NA | -3.723786e-04 | + | NA | NA | + | + | -160.6231 | 11.46623748 | F9 |
| 0.53047658 | NA | -3.475335e-04 | NA | -0.124655717 | 0.0009291314 | + | + | -160.5474 | 11.54192259 | F9 |
| 0.67185384 | NA | NA | + | NA | NA | + | + | -160.3708 | 11.71852642 | F9 |
| 0.77016320 | -4.147112e-04 | -3.181135e-04 | + | -0.628643763 | NA | + | + | -159.7104 | 12.37896517 | F9 |
| 0.73547477 | NA | -3.690328e-04 | + | NA | 0.0027192299 | + | + | -159.3642 | 12.72511113 | F9 |
| 0.64701297 | NA | NA | + | NA | 0.0046838733 | + | + | -159.3504 | 12.73896082 | F9 |
| 0.72763771 | -5.069862e-04 | NA | + | -0.659425738 | -0.0044795905 | + | + | -159.2950 | 12.79439999 | F9 |
| 0.79758786 | -4.535390e-04 | -3.206138e-04 | + | -0.622586989 | -0.0052827496 | + | + | -157.9219 | 14.16747644 | F9 |
| 0.78865478 | NA | -3.615461e-04 | + | -0.619600683 | NA | + | + | -156.4995 | 15.58983142 | F9 |
| 0.71511659 | NA | NA | + | -0.664435847 | NA | + | + | -155.8328 | 16.25654112 | F9 |
| 0.77262618 | NA | -3.582062e-04 | + | -0.618078076 | 0.0028959539 | + | + | -155.3122 | 16.77711442 | F9 |
| 0.68971370 | NA | NA | + | -0.663052706 | 0.0047797706 | + | + | -154.9028 | 17.18654261 | F9 |
| 0.62551041 | NA | -4.539630e-04 | NA | NA | NA | + | + | -433.7438 | 0.00000000 | F10 |
| 0.63063908 | NA | -4.593714e-04 | NA | NA | -0.0007632462 | + | + | -432.2543 | 1.48952605 | F10 |
| 0.61425762 | NA | -4.407719e-04 | NA | 0.181135169 | NA | + | + | -432.0142 | 1.72954079 | F10 |
| 0.62410602 | -2.130360e-04 | -3.831129e-04 | NA | NA | NA | + | + | -431.7195 | 2.02428231 | F10 |
| 0.61743645 | NA | -4.446750e-04 | NA | 0.179597066 | -0.0004433790 | + | + | -430.5759 | 3.16791917 | F10 |
| 0.61610271 | -1.958731e-04 | -3.800040e-04 | NA | 0.132864001 | NA | + | + | -430.2730 | 3.47077158 | F10 |
| 0.64077577 | -2.200138e-04 | -3.957285e-04 | NA | NA | -0.0025647971 | + | + | -429.9650 | 3.77877214 | F10 |
| 0.55281369 | NA | NA | NA | NA | NA | + | + | -429.3677 | 4.37605872 | F10 |
| 0.63056777 | -2.022551e-04 | -3.911723e-04 | NA | 0.127882573 | -0.0021715013 | + | + | -428.5998 | 5.14399114 | F10 |
| 0.53956117 | NA | NA | NA | 0.262624879 | NA | + | + | -427.5614 | 6.18237266 | F10 |
| 0.50773455 | NA | NA | NA | NA | 0.0080717495 | + | + | -427.3474 | 6.39638164 | F10 |
| 0.56978277 | -3.636333e-04 | NA | NA | NA | NA | + | + | -426.4968 | 7.24696757 | F10 |
| 0.49353088 | NA | NA | NA | 0.265748188 | 0.0082138411 | + | + | -425.4472 | 8.29659937 | F10 |
| 0.54791895 | -3.457163e-04 | NA | NA | NA | 0.0037651665 | + | + | -425.4213 | 8.32245956 | F10 |
| 0.56058084 | -3.411642e-04 | NA | NA | 0.161575934 | NA | + | + | -425.0470 | 8.69677269 | F10 |
| 0.53624778 | -3.208739e-04 | NA | NA | 0.167756441 | 0.0041316266 | + | + | -423.9563 | 9.78750525 | F10 |
| 0.66268338 | NA | -4.052012e-04 | + | NA | NA | + | + | -419.4652 | 14.27859934 | F10 |
| 0.64729337 | -2.192566e-04 | -3.485131e-04 | + | NA | NA | + | + | -418.7321 | 15.01168947 | F10 |
| 0.65164353 | NA | -4.045073e-04 | + | 0.094484792 | NA | + | + | -418.5005 | 15.24329684 | F10 |

| Intercept | Dist. Forest edge | Dist. Roads | Land cover | *Proportion developed* | *Landscape heterogeneity* | s(Space) | s(Time of day) | AICc | ΔAIC | Indiv. |
|---|---|---|---|---|---|---|---|---|---|---|
| 0.64903746 | *NA* | -3.939662e-04 | + | *NA* | 0.0020880829 | + | + | -417.9738 | 15.76993716 | F10 |
| 0.63949580 | -2.149203e-04 | -3.501711e-04 | + | 0.070583881 | *NA* | + | + | -417.7889 | 15.95487883 | F10 |
| 0.64984814 | -2.211906e-04 | -3.519824e-04 | + | *NA* | -0.0003938543 | + | + | -417.1003 | 16.64348622 | F10 |
| 0.63772092 | *NA* | -3.931119e-04 | + | 0.095191348 | 0.0021275458 | + | + | -417.0360 | 16.70781275 | F10 |
| 0.64136446 | -2.163072e-04 | -3.530649e-04 | + | 0.070180838 | -0.0002723475 | + | + | -416.1773 | 17.56648683 | F10 |
| 0.59258318 | *NA* | *NA* | + | *NA* | *NA* | + | + | -413.4978 | 20.24601893 | F10 |
| 0.58430412 | -3.590319e-04 | *NA* | + | *NA* | *NA* | + | + | -413.0596 | 20.68417377 | F10 |
| 0.57651192 | *NA* | *NA* | + | 0.142155113 | *NA* | + | + | -412.8532 | 20.89059492 | F10 |
| 0.57387021 | -3.526058e-04 | *NA* | + | 0.091858534 | *NA* | + | + | -412.2923 | 21.45143396 | F10 |
| 0.55274796 | -3.190384e-04 | *NA* | + | *NA* | 0.0059033633 | + | + | -411.8516 | 21.89213217 | F10 |
| 0.53393972 | *NA* | *NA* | + | *NA* | 0.0108349351 | + | + | -411.1298 | 22.61394957 | F10 |
| 0.54162011 | -3.125506e-04 | *NA* | + | 0.095421272 | 0.0059665406 | + | + | -411.0738 | 22.66994326 | F10 |
| 0.51886362 | *NA* | *NA* | + | 0.137728403 | 0.0107104621 | + | + | -410.4042 | 23.33959264 | F10 |
| 0.37447853 | *NA* | *NA* | *NA* | *NA* | 0.0187873591 | + | + | -160.8217 | 0.00000000 | F11 |
| 0.46747139 | *NA* | *NA* | *NA* | *NA* | *NA* | + | + | -160.2879 | 0.53384267 | F11 |
| 0.37348906 | *NA* | *NA* | *NA* | 0.383157836 | 0.0187480617 | + | + | -160.1996 | 0.62215738 | F11 |
| 0.46626990 | *NA* | *NA* | *NA* | 0.388822889 | *NA* | + | + | -159.7585 | 1.06322325 | F11 |
| 0.40580580 | -2.473261e-04 | *NA* | *NA* | *NA* | 0.0162747424 | + | + | -158.4484 | 2.37331699 | F11 |
| 0.49099957 | -3.080466e-04 | *NA* | *NA* | *NA* | *NA* | + | + | -157.8774 | 2.94431138 | F11 |
| 0.40458063 | -2.466527e-04 | *NA* | *NA* | 0.382415594 | 0.0162731355 | + | + | -157.8052 | 3.01649818 | F11 |
| 0.47402513 | *NA* | -3.887832e-04 | *NA* | *NA* | 0.0182786774 | + | + | -157.5016 | 3.32013350 | F11 |
| 0.48972784 | -3.069124e-04 | *NA* | *NA* | 0.383517737 | *NA* | + | + | -157.2917 | 3.53007672 | F11 |
| 0.56659392 | *NA* | -3.971727e-04 | *NA* | *NA* | *NA* | + | + | -157.2660 | 3.55576368 | F11 |
| 0.47002998 | *NA* | -3.763295e-04 | *NA* | 0.300988276 | 0.0182699920 | + | + | -156.7696 | 4.05213053 | F11 |
| 0.56252511 | *NA* | -3.845972e-04 | *NA* | 0.301074787 | *NA* | + | + | -156.5727 | 4.24907995 | F11 |
| 0.49072772 | -1.923520e-04 | -3.560044e-04 | *NA* | *NA* | 0.0162196674 | + | + | -155.4853 | 5.33647004 | F11 |
| 0.57544190 | -2.533798e-04 | -3.550811e-04 | *NA* | *NA* | *NA* | + | + | -155.1553 | 5.66643805 | F11 |
| 0.48655303 | -1.930209e-04 | -3.430866e-04 | *NA* | 0.306919890 | 0.0162304669 | + | + | -154.7417 | 6.08007156 | F11 |
| 0.57129609 | -2.537584e-04 | -3.421441e-04 | *NA* | 0.306159912 | *NA* | + | + | -154.4304 | 6.39135689 | F11 |
| 0.40351119 | *NA* | *NA* | + | *NA* | *NA* | + | + | -152.8895 | 7.93225026 | F11 |
| 0.42261014 | -6.629329e-04 | *NA* | + | *NA* | *NA* | + | + | -152.5638 | 8.25795670 | F11 |
| 0.38185832 | *NA* | *NA* | + | 0.453381512 | *NA* | + | + | -152.4158 | 8.40594082 | F11 |
| 0.31765350 | -5.509065e-04 | *NA* | + | *NA* | 0.0212919561 | + | + | -151.9512 | 8.87050991 | F11 |
| 0.39635701 | -6.625842e-04 | *NA* | + | 0.436050033 | *NA* | + | + | -151.7950 | 9.02677494 | F11 |
| 0.27582894 | *NA* | *NA* | + | *NA* | 0.0271734420 | + | + | -151.4304 | 9.39137747 | F11 |
| 0.28860934 | -5.512417e-04 | *NA* | + | 0.442395112 | 0.0214213942 | + | + | -151.0311 | 9.79061412 | F11 |
| 0.25061213 | *NA* | *NA* | + | 0.444069748 | 0.0271918161 | + | + | -150.6592 | 10.16252661 | F11 |
| 0.48381476 | -6.613839e-04 | -4.067244e-04 | + | *NA* | *NA* | + | + | -150.5929 | 10.22879084 | F11 |
| 0.46575446 | *NA* | -3.961001e-04 | + | *NA* | *NA* | + | + | -150.0446 | 10.77713739 | F11 |
| 0.38334671 | -5.580842e-04 | -3.657449e-04 | + | *NA* | 0.0188766147 | + | + | -149.9352 | 10.88655579 | F11 |
| 0.45570559 | -6.618689e-04 | -3.961460e-04 | + | 0.379668879 | *NA* | + | + | -149.6643 | 11.15741279 | F11 |
| 0.44204412 | *NA* | -3.839882e-04 | + | 0.390710500 | *NA* | + | + | -149.4146 | 11.40712301 | F11 |
| 0.35184082 | -5.582883e-04 | -3.547820e-04 | + | 0.394558443 | 0.0191013834 | + | + | -148.9501 | 11.87166299 | F11 |
| 0.33719993 | *NA* | -3.496370e-04 | + | *NA* | 0.0255412184 | + | + | -148.7621 | 12.05958963 | F11 |
| 0.30944127 | *NA* | -3.384725e-04 | + | 0.396643815 | 0.0256536008 | + | + | -147.9206 | 12.90117424 | F11 |
| 0.51887994 | -2.883929e-04 | *NA* | + | *NA* | 0.0279665524 | + | + | -539.3394 | 0.00000000 | F12 |
| 0.50893533 | -2.972261e-04 | *NA* | + | -0.912479939 | 0.0284812892 | + | + | -537.8103 | 1.52907268 | F12 |
| 0.53783006 | -2.588108e-04 | -8.832322e-05 | + | *NA* | 0.0267753179 | + | + | -537.4385 | 1.90096230 | F12 |
| 0.52836996 | -2.666986e-04 | -9.195519e-05 | + | -0.940249892 | 0.0272591453 | + | + | -535.9759 | 3.36353334 | F12 |
| 0.52977837 | *NA* | *NA* | + | *NA* | 0.0287165145 | + | + | -535.8267 | 3.51272277 | F12 |
| 0.56338547 | *NA* | -1.656126e-04 | + | *NA* | 0.0262895522 | + | + | -534.4277 | 4.91174320 | F12 |
| 0.52243584 | *NA* | *NA* | + | -0.700858512 | 0.0291322073 | + | + | -533.3254 | 6.01398958 | F12 |
| 0.55611931 | *NA* | -1.706029e-04 | + | -0.790172338 | 0.0266904318 | + | + | -532.0185 | 7.32093340 | F12 |
| 0.66127207 | -3.190726e-04 | *NA* | + | *NA* | *NA* | + | + | -531.2864 | 8.05303559 | F12 |
| 0.65699706 | -3.246170e-04 | *NA* | + | -0.528070725 | *NA* | + | + | -529.4558 | 9.88364576 | F12 |
| 0.69058520 | -2.447495e-04 | -2.022128e-04 | + | *NA* | *NA* | + | + | -529.0335 | 10.30593596 | F12 |
| 0.68609880 | -2.499804e-04 | -2.057972e-04 | + | -0.622787618 | *NA* | + | + | -527.3068 | 12.03262684 | F12 |
| 0.67777476 | *NA* | *NA* | + | *NA* | *NA* | + | + | -525.6584 | 13.68103647 | F12 |
| 0.71229632 | *NA* | -2.740815e-04 | + | *NA* | *NA* | + | + | -525.3308 | 14.00863906 | F12 |
| 0.67563873 | *NA* | *NA* | + | -0.286734507 | *NA* | + | + | -523.2276 | 16.11183609 | F12 |
| 0.70918890 | *NA* | -2.780392e-04 | + | -0.485806990 | *NA* | + | + | -522.8863 | 16.45312770 | F12 |
| 0.26933204 | -3.245694e-04 | *NA* | *NA* | *NA* | 0.0280434768 | + | + | -522.6928 | 16.64658072 | F12 |
| 0.26711953 | -3.297459e-04 | *NA* | *NA* | -0.793880709 | 0.0285187145 | + | + | -521.0674 | 18.27204765 | F12 |

| Intercept | Dist. Forest edge | Dist. Roads | Land cover | *Proportion developed* | *Landscape heterogeneity* | s(Space) | s(Time of day) | AICc | ΔAIC | Indiv. |
|---|---|---|---|---|---|---|---|---|---|---|
| 0.30152924 | -2.716365e-04 | -1.482130e-04 | *NA* | *NA* | 0.0261960257 | + | + | -520.3043 | 19.03513623 | F12 |
| 0.30005825 | -2.757050e-04 | -1.524262e-04 | *NA* | -0.849844219 | 0.0266555479 | + | + | -518.7741 | 20.56531774 | F12 |
| 0.25492832 | *NA* | *NA* | *NA* | *NA* | 0.0288054423 | + | + | -516.8569 | 22.48250406 | F12 |
| 0.31395511 | *NA* | -2.517756e-04 | *NA* | *NA* | 0.0253959181 | + | + | -515.7086 | 23.63086136 | F12 |
| 0.25305306 | *NA* | *NA* | *NA* | -0.597815848 | 0.0291788266 | + | + | -514.4302 | 24.90919148 | F12 |
| 0.31275826 | *NA* | -2.568022e-04 | *NA* | -0.744326190 | 0.0257998544 | + | + | -513.7614 | 25.57800066 | F12 |
| 0.42935655 | -3.433184e-04 | *NA* | *NA* | *NA* | *NA* | + | + | -513.3544 | 25.98499686 | F12 |
| 0.42960529 | -3.461001e-04 | *NA* | *NA* | -0.392097660 | *NA* | + | + | -511.3998 | 27.93964078 | F12 |
| 0.46633484 | -2.484470e-04 | -2.535567e-04 | *NA* | *NA* | *NA* | + | + | -510.7751 | 28.56432985 | F12 |
| 0.46718321 | -2.508920e-04 | -2.570774e-04 | *NA* | -0.526671840 | *NA* | + | + | -508.9397 | 30.39969446 | F12 |
| 0.47308685 | *NA* | -3.454051e-04 | *NA* | *NA* | *NA* | + | + | -505.8649 | 33.47453662 | F12 |
| 0.41870324 | *NA* | *NA* | *NA* | *NA* | *NA* | + | + | -505.3452 | 33.99417342 | F12 |
| 0.47383732 | *NA* | -3.490281e-04 | *NA* | -0.434642888 | *NA* | + | + | -503.7627 | 35.57676665 | F12 |
| 0.41877548 | *NA* | *NA* | *NA* | -0.174410412 | *NA* | + | + | -503.0267 | 36.31267011 | F12 |
